# *Mesobuthus* Venom-Derived Antimicrobial Peptides Possess Intrinsic Multifunctionality and Differential Potential as Drugs

**DOI:** 10.3389/fmicb.2018.00320

**Published:** 2018-02-27

**Authors:** Bin Gao, Shunyi Zhu

**Affiliations:** Group of Peptide Biology and Evolution, State Key Laboratory of Integrated Management of Pest Insects and Rodents, Institute of Zoology, Chinese Academy of Sciences, Beijing, China

**Keywords:** peptide antibiotics, venom gland immunity, *Staphylococci*, predation, defense

## Abstract

Animal venoms are a mixture of peptides and proteins that serve two basic biological functions: predation and defense against both predators and microbes. Antimicrobial peptides (AMPs) are a common component extensively present in various scorpion venoms (herein abbreviated as svAMPs). However, their roles in predation and defense against predators and potential as drugs are poorly understood. Here, we report five new venom peptides with antimicrobial activity from two *Mesobuthus* scorpion species. These α-helical linear peptides displayed highly bactericidal activity toward all the Gram-positive bacteria used here but differential activity against Gram-negative bacteria and fungi. In addition to the antibiotic activity, these AMPs displayed lethality to houseflies and hemotoxin-like toxicity on mice by causing hemolysis, tissue damage and inducing inflammatory pain. Unlike AMPs from other origins, these venom-derived AMPs seem to be unsuitable as anti-infective drugs due to their high hemolysis and low serum stability. However, MeuTXKβ1, a known two-domain *Mesobuthus* AMP, is an exception since it exhibits high activity toward antibiotic resistant *Staphylococci* clinical isolates with low hemolysis and high serum stability. The findings that the classical AMPs play predatory and defensive roles indicate that the multifunctionality of scorpion venom components is an intrinsic feature likely evolved by natural selection from microbes, prey and predators of scorpions. This definitely provides an excellent system in which one can study how a protein adaptively evolves novel functions in a new environment. Meantimes, new strategies are needed to remove the toxicity of svAMPs on eukaryotic cells when they are used as leads for anti-infective drugs.

## Introduction

The venoms of various animals (e.g., scorpions, cone snails, snakes, spiders, sea anemones, and lizards) are produced in specialized glands that are associated with a delivery device, by which the compound can be directly introduced into the body of a recipient (Brodie, 2009). Venoms serve two basic biological functions: predation (food acquisition) and defense against competitors and microbes in the host bodies and environments (Brodie, 2009; Otti et al., 2014). Scorpion venoms are typically cocktails of various toxic peptide and protein constituents. Studies on their chemical nature have identified peptide neurotoxins affecting ion channels as a major toxic ingredient. In addition, scorpion venoms contain a variety of relatively low-abundant antimicrobial peptides (AMPs) that are active on a wide range of microbes, such as bacteria, fungi, viruses, and protozoans (Zeng et al., 2005; du Plessis et al., 2008; Almaaytah and Albalas, 2014; Harrison et al., 2014; Ortiz et al., 2015). These scorpion venom AMPs (svAMPs) are characterized by biochemical purification from the venoms and molecular cloning from the venom glands. When purified from the venoms by reversed-phase high-performance liquid chromatography (RP-HPLC), they are usually eluted following K^+^ and Na^+^ channel toxins in a C_18_ column (Conde et al., 2000; Torres-Larios et al., 2000; Corzo et al., 2001; Moerman et al., 2002; this work), indicative of their higher hydrophobicity. This method has led to the discovery of an array of svAMPs from various scorpion venoms (e.g., Hadrurin from *Hadrurus aztecus*, Parabutoporin from *Parabuthus schlechteri*, and Scorpine from *Pandinus imperator*; Conde et al., 2000; Torres-Larios et al., 2000; Remijsen et al., 2010). Many more svAMPs were found by cDNA cloning followed by functional identification with synthetic peptides (e.g., Meucin-13, Meucin-18, Meucin-24, and Meucin-25 from *Mesobuthus eupeus*; Mucroporin from *Lychas Mucronatus*, Imcroporin from *Isometrus maculates*, and Ctriporin from *Chaerilus tricostatus*, etc., Dai et al., 2008; Gao et al., 2009, 2010; Zhao et al., 2009; Fan et al., 2011).

Since the discovery of the first svAMPs in 2000, more than 70 such peptides have been characterized so far, the majority of which are cationic and consist of 13–84 residues (Zeng et al., 2005; du Plessis et al., 2008; Almaaytah and Albalas, 2014; Harrison et al., 2014; Ortiz et al., 2015). Structurally, they form two distinct categories: (1) svAMPs belonging to the non-disulfide-bridged peptides (NDBPs). This class of molecules adopts a random coil configuration in aqueous solution but folds into an α-helical structure in a membrane mimicking environment. According to their molecular sizes, Strong and colleagues further divide them into three subclasses (Harrison et al., 2014): (a) Long-chain peptides of 41–56 amino acids, such as Hadrurin from *Hadrurus aztecus*, Parabutoporin from *Parabuthus schlechteri*, Pandinin 1 from *Pandinus imperator*, and Opistoporin 1 and 2 from *Opistophtalmus carinatus* (Torres-Larios et al., 2000; Corzo et al., 2001; Moerman et al., 2002; Remijsen et al., 2010); (b) Intermediate-chain peptides of 24–29 amino acids, such as Meucin-24, Meucin-25, Pandinin 2 from *Pandinus imperator* (Corzo et al., 2001; Gao et al., 2009); and (c) Short-chain peptides of 13–19 amino acids, such as Meucin-13, Meucin-18, Mucroporin, Imcroporin, Ctriporin, StCT1 from *Scorpiops tibetanus*, and IsCT and IsCT2 from *Opisthacanthus madagascariensis* (Dai et al., 2001, 2008; Gao et al., 2009; Zhao et al., 2009; Yuan et al., 2010; Fan et al., 2011; Harrison et al., 2014); (2) svAMPs containing the CSαβ structural motif. This class of peptides are usually composed of 58-84 amino acids that include two distinct families: (a) The βSPN (β-KTxs and Scorpines) family. Members in this family are composed of an N-terminal α-helical domain (NHD) and a C-terminal CSαβ domain (CCD). The former is organized as a helix-hinge-helix structure and the latter resembles an ancient invertebrate-type defensin (AITD). Similar organization is also present in big defensins (Kouno et al., 2009). Whereas scorpines and β-KTxs both are toxic to microorganisms, only β-KTxs have some activity on mammalian K^+^ channels (Conde et al., 2000; Zhu and Tytgat, 2004; Diego-García et al., 2008; Zhu et al., 2010); (b) Scorpion β-toxin-like peptides, including Bactridines 1–6 from *Tityus discrepans* and Cm38 from *Centruroides margaritatus* (Díaz et al., 2009; Dueñas-Cuellar et al., 2015). These peptides share high sequence similarity to β-toxins, and some of them have been identified to have ability to shift the voltage dependence of activation of voltage-gated Na^+^ (Na_v_) channels to more negative membrane potentials, a typical β-effect (Peigneur et al., 2012). Given that these peptides possess some neurotoxicity on ion channels, we call them scorpion venom neurotoxin-type AMPs (abbreviated as svntAMPs), and accordingly the α-helical peptides are called scorpion venom classical AMPs (abbreviated as svcAMPs) in view of their structural and functional similarity to some typical AMPs from insects (e.g., Cecropin), frogs (e.g., Magainin) and vertebrates (e.g., LL-37; Boman and Hultmark, 1987; Zasloff, 1987; Zhu and Gao, 2017).

In the present work, we conducted a systematic survey on svAMPs from *Mesobuthus eupeus* and its sibling species *M. martensii*. The objectives were: (1) to identify new types of svAMPs; (2) to assay the antimicrobial spectra of svAMPs; (3) to explore the biological roles of svAMPs in both predation and defense; (4) to evaluate the therapeutic potential of svAMPs as peptide antibiotics. This work reveals: (1) a diversity of svAMPs from *Mesobuthu*s and their broad-spectrum antibacterial activity; (2) the defensive role of svcAMPs against scorpions' predators via acting as hemotoxins to induce pain; (3) the insecticidal toxicity of svcAMPs likely involved in scorpions' predation; (4) the therapeutic potential of MeuTXKβ1, a two-domain svntAMP from the *Mesobuthus eupeus* venom.

## Materials and methods

### Ethics statement

All experimental protocols related to animals (mice, pigeons, and lizards) used in this study were reviewed and approved by the Animal Care and Use Committee of Institute of Zoology, the Chinese Academy of Sciences. The institute does not issue a protocol number to any animal study, but each study requires the permit to use animals from the ethical committee. The animal facility must be licensed by the experimental animal committee of Beijing, and all staff, fellows and students must receive appropriate training before performing animal studies.

### Experimental animals

*Mesobuthus eupeus* and *M. martensiis* were collected from Ningxia and Beijing (China), respectively. Adult houseflies (*Musca domestica*) were provided by Prof. X. Qiu. Mice were purchased from the Experimental Animal Center, the Academy of Military Medical Sciences (Beijing, China) and housed in a temperature controlled room (22–25°C) with water and food available *ad libitum*, and on a 12 h light/dark cycle.

### Isolation of cDNA and genomic clones

Total RNA and genomic DNA (gDNA) were extracted from the venom gland and legs of scorpions, respectively, according to the methods described previously (Zhu and Gao, 2006). The total RNA was dissolved in H_2_O and used for reverse-transcribed into the first strand cDNA with RT-PreMix Kit and a universal oligo(dT)-containing adaptor primer (dT3AP). The cDNA was used for PCR amplification of genes of interest by a specific forward primer and a universal reverse primer (3AP). Primer sequences are provided in Table S1 (Supplementary Materials). The extracted gDNA was directly used as template for PCR by two specific primers. PCR products were ligated into pGM-T and resultant recombinant plasmids were transformed into *Escherichia coli* DH5α. Recombinant clones were sequenced through the chain termination method by T7 or SP6. Nucleotide sequences for the genes reported here have been deposited in the GenBank database (http://www.ncbi.nlm.nih.gov/) under accession numbers provided in Table S2. Other than PCR, the venom gland cDNA libraries of *M. martensii* and *M. eupeus* previously constructed (Zhu et al., 1999, 2011) were also used to isolate cDNA clones of some svAMPs (Table S2).

### Biochemical isolation and chemical synthesis of svAMPs

For isolation of svAMPs, the *M. eupeus* venom was collected by electrical stimulation with the Multipurpose Instrument in Pharmacology and Physiology (YSD-4G) (Zhenghua Biological Instrument Co., Anhui, China; Gao et al., 2007). From six scorpions, we collected a total of 12 μl of crude venom (~2 μl each) that was suspended in 500 μl of 0.05% trifluoroacetic acid (TFA, v/v) and directly subjected to reversed-phase high-performance liquid chromatography (RP-HPLC) following centrifugation at 12,000 g for 30 min at 4°C (Zhu et al., 2012b). The Agilent Zorbax 300SB-C18 (4.6 × 150 mm, 5 μm) was equilibrated with 0.05% TFA in water (v/v) and peptide components were eluted from the column with a linear gradient from 0 to 60% acetonitrile in 0.05% TFA in water (v/v) within 60 min with a flow rate of 1 ml/min. The UV absorbance trace was followed at a wavelength of 225 nm. All well-defined peaks were collected separately and lyophilized by Thermo Scientific SAVANT SPD1010 SpeedVac Concentrator (USA). Lyophilized peptides were dissolved in water and their concentrations were determined according to the Bradford method (Bradford, 1976). Purity and molecular mass of peptides were determined by matrix-assisted laser desorption/ionization time of flight mass spectrometry (MALDI-TOF MS) on ultrafleXtreme MALDI-TOF/TOF Mass Spectrometer (Bruker, USA). N-terminal sequences of proteins were determined by automated Edman degradation on the PPSQ-31A protein sequencer (Shimadzu, Kyoto, Japan). The α-helical svAMPs deduced from their genes were chemically synthesized in ChinaPeptides Co., Ltd. (Shanghai) with purity >95%, as confirmed by RP-HPLC and MALDI-TOF MS.

### Circular dichroism (CD) spectroscopy

The CD spectra of six linear AMPs (MeuFSPL-1, MeuFSPL-2, Marmelittin, Melittin, Meucin-22, and Marcin-22) were recorded on the Chirascan Plus spectropolarimeter (UK) at a protein concentration of 0.1 mg/mL dissolved in H_2_O or 50% trifluoroethanol (TFE). For comparison with the recombinant version, the native MeuTXKβ1 was also analyzed by circular dichroism spectroscopy. Fifty percent trifluoroethanol was employed to provide a membrane-mimicking environment to study structural change of the linear peptides in contact with bacterial membrane (Gao et al., 2009). Spectra were measured at 20°C from 260 to 190 nm with a quartz cell of 1.0 mm thickness and data are expressed as mean residue molar ellipticity ([θ]), calculated as follows: [θ] = θx(0.1xMRW)/(LxC), where θ is the ellipticity (in millidegrees), C is the concentration (in mg/mL), L is the path-length (in cm), and MRW is the mean residue weight (in Da).

### Antimicrobial assay

The antimicrobial activity of seven peptides, including MeuFSPL-1, MeuFSPL-2, Marmelittin, Melittin, Meucin-22, Marcin-22, and MeuTXKβ1, was evaluated by the inhibition-zone assay previously described in our studies (Hultmark, 1998; Zhu et al., 2012a; Zhu and Gao, 2014). Temperatures used were 30 or 37°C for bacteria or 28°C for fungi. Media used were Luria-Bertani (LB) Broth (10 g tryptone, 5 g yeast extract, and 5 g NaCl in 1 l of water) or Nutrient Broth (10 g peptone, 5 g beef extract powder, and 5 g NaCl in 1 l of water) or GYM(Isp2) (4 g yeast extract, 10 g malt extract, 4 g glucose, 2 g CaCO_3_ in 1 l of water) for bacteria or Potato Dextrose Broth (200 g patato and 20 g glucose in 1 l of water) for fungi. To make plates, 10 μl of microbial culture (~2 × 10^6^ bacterial cells or fungal spores/ml) were mixed in 6 ml of medium containing 0.8% agar and poured into Peri dishes of 9.0 cm diameter. Wells with a diameter of 2 mm were punched into the medium, filled with 2 μl of sample each well. In this assay, three different doses of peptides were applied to three independent wells in one standard bacterial plate. After incubation overnight, inhibition zones were measured and used to calculate lethal concentrations (C_*L*_), a concentration just sufficient to inhibit bacterial growth (Hultmark, 1998; Gao et al., 2009). C_*L*_ values are calculated from a plot of d^2^ against log n, where d is the diameter (in cm) and n is the amount of sample applied in the well (in nmol). The plot is linear and thus C_*L*_ can be calculated from the slope (k) and the intercept (m) of this plot. The formula used here is C_*L*_ = 2.93/ak10^m/k^, where “a” is the thickness of the bacterial plate and “C_*L*_” is in μM. This is a quantitative description of the qualitative inhibition zone assay since the days of Alexander Fleming and in most cases the calculated C_*L*_ are comparable to the directly measured minimal inhibitory concentrations (MIC) via broth micro-dilution assay (Hultmark, 1998). The sources of microbes used in this study are listed in Table S3.

### Killing kinetics

The *in vitro* killing curve for MeuTXKβ1 was determined against the bacterium *S. aureus* PRSA P1383. For the assay, a single fresh colony was inoculated into LB (Luria-Bertani) medium (10 g tryptone, 5 g yeast extract, and 5 g NaCl in 1 l of H_2_O) and incubated at 37°C until OD_600_ of 0.2. The initial concentration used was 4 × 10^7^ cells/ml. Samples for colony counts were taken at time 0, 5, 10, 15, 20, 30, and 60 min after addition of peptides. Results are given as colony-forming units (CFUs) per milliliter.

### Membrane permeability assay

To assess the permeation ability of MeuTXKβ1 on bacterial membrane, about 5 × 10^5^ bacterial cells (*Staphylococcus aureus* PRSA P1383) in 500 μL of PBS (140 mM NaCl, 2.7 mM KCl, 10 mM Na_2_HPO_4_, 1.8 mM KH_2_PO_4_, pH 7.3) were mixed with 1 μM propidium iodide (PI) for 5 min in the dark. Fluorescence was measured with the F-4500 FL spectrophotometer (Hitachi High-Technology Company). Once the basal fluorescence reached a constant value, peptides at 5 × C_*L*_ were added, and changes in fluorescence arbitrary were monitored (λ_exc_ = 525 nm; λ_ems_ = 595 nm).

### Scanning electron microscope

Bacterial cells (*S. aureus* PRSA P1383) at the exponential growth phase were treated with MeuTXKβ1 at 5 × C_*L*_ at 37°C for 90 min. After centrifugation, bacterial pellets were fixed with 2.5% glutaraldehyde for 1 h, followed by washing three times with PBS. Dehydration was carried out with a series of graded ethanol solution. Cells were then dried by BAL-TEC CPD030 critical point dryer before being mounted on carbon tape, sputtered with gold coating (BAL-TEC SCD005). Images were visualized in FEI QUANTA 200.

### Hemolytic assay

Hemolytic activity of peptides against fresh erythrocytes from the ICR mice (*Mus musculus*), the lizards (*Eremias multiocellata*) or the pigeons (*Columba livia* domestica) was assayed according to the standard method (Zhu et al., 2012a). An aliquot of freshly prepared venom was diluted with 0.9% NaCl to a protein concentration of ~5 mg/mL for use. The percentage of hemolysis is determined as (A_pep_-A_blank_)/(A_tot_-A_blank_) × 100, in which “A” represents absorbance measured at 570 nm. A_blank_ and A_prep_ were evaluated in the absence or presence of the venom or peptides. 100% hemolysis (A_tot_) was obtained in the presence of 1% Triton X-100.

### Serum stability

To assess serum stability, peptides were dissolved in H_2_O or fresh mouse serum for the indicated times at 37°C and their residual activity was measured by the inhibition-zone assay (Hultmark, 1998).

### Peptide-induced pain test

Healthy male ICR (imprinting control region) mice, weighing 18–22 g, were used in this study. Ten microgram of marmelittin dissolved in 50 μl of 0.9% NaCl was injected into the right hinder sole of mice. Immediately after peptide injection, mice were individually placed in a glass observation chamber with a transparent floor. Licking times were recorded when animals began to frequently lick the injected paws. Melittin and 0.9% NaCl were used as positive and negative controls.

### Insect toxicity assays

Insect toxicity of peptides were assayed according to the method of Maggio and King (26). Insect saline contains 156 mM NaCl, 7 mM KCl, 8 mM CaCl_2_, 4 mM MgCl_2_ (pH 7.0). Peptide solutions were diluted in insect saline. One mircorliter of peptide solution at different doses was injected into each housefly adult (about 10 mg) via micro-injector and flies injected with insect saline were used as control. Ten to fifteen flies were used for each group, and experiments were performed in triplication. Dead individuals were recorded at 24 h post-injection and half maximal lethal dose (LD_50_) values were calculated from the dose-response data by GraphPad Prism 5.

### Sequence and structural analyses

Sequences were aligned by ClustalX (http://www.clustal.org/) and helical wheel projections were generated at the server (http://rzlab.ucr.edu/scripts/wheel/wheel.cgi). Three-dimensional (3D) structures of marmelittin and Meucin-22 were built by comparative modeling (Ginalski, 2006) at Swiss-Model, a fully automated protein structure homology-modeling serve (http://www.expasy.ch/swissmod/SWISS-MODEL.html). Initial backbone fitting and energy minimization steps were performed with the DeepView program (http://www.expasy.ch/spdbv) and further refinement was achieved via submission to the Swiss-Model server. The model quality was evaluated by Verify3D (Eisenberg et al., 1997). Hydropathy plots were performed by ProtScale (http://web.expasy.org/protscale/). Scores > or < 0 represent hydrophobicity or hydrophilicity.

### Statistical analysis

Unpaired two-tailed Student's *t*-test was used to compare means between control and the treatment group with SPSS (SPSS Inc.). ^*^P < 0.05; ^**^P < 0.01; ^***^P < 0.001.

## Results

### svcAMPs: structurally homologous to frog skin AMPs

**Figure 1:**
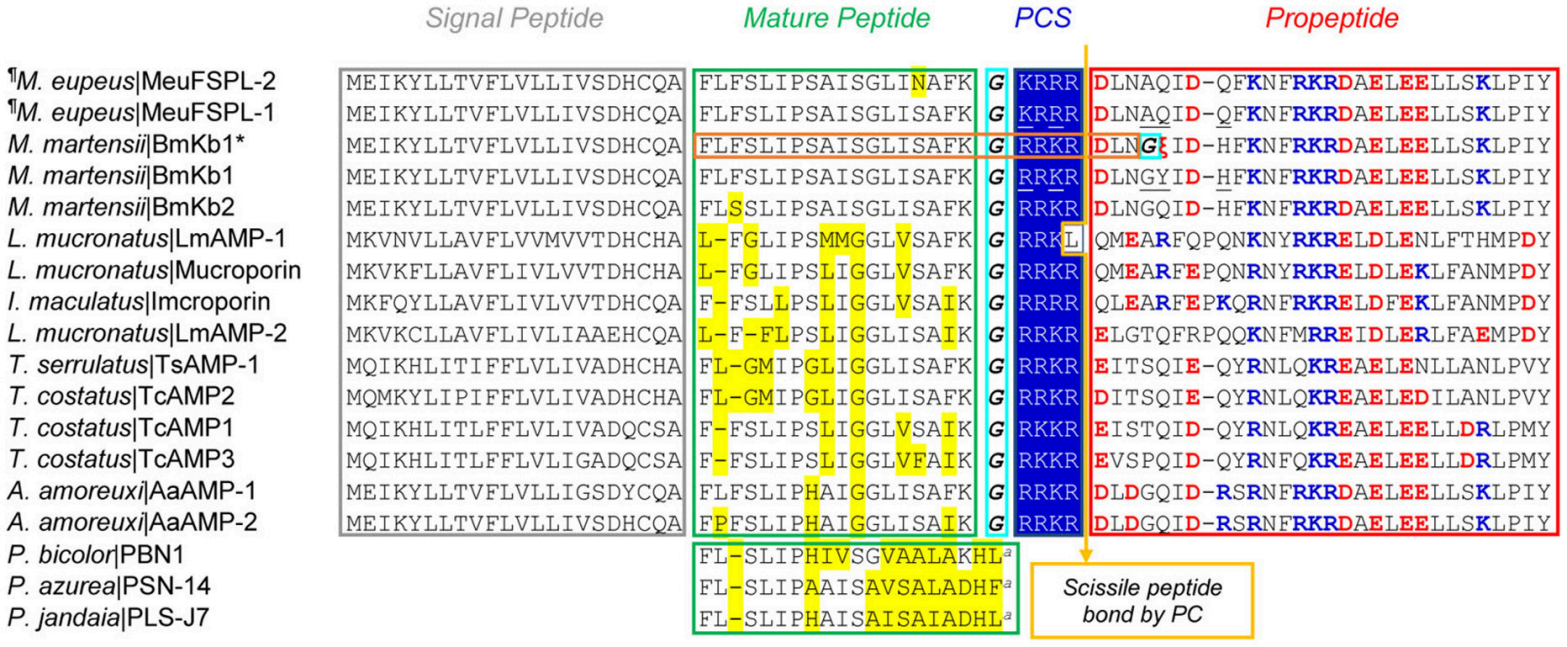
MeuFSPLs and homologs. For each peptide, scorpion species and peptide names are shown. Glycines for C-terminal amidation are italicized and boxed in *cyan*. PCS, proprotein convertase signal. The arrow represents scissile peptide bond by PC. In the mature region, residues non-identical to MeuFSPL-1 are shadowed in *yellow*, and in the proregion, charged residues are colored (basic, *blue*; acidic, *red*). “^a^,” amidation. Non-identical residues between MeuFSPL-1 and BmKb1 are underlined once. Except three from frogs (*Phyllobates azzurea, P. bicolor*, and *P. jandaia*), all other sequences are derived from scorpions (*Androctonus amoreuxi, Isometrus maculatus, Lychas mucronatus, Mesobuthus eupeus, Mesobuthus martensii, Tityus costatus*, and *Tityus serrulatus*). These sequences can be retrieved from GenBank. “^¶^” represents new sequences reported in this work. The proposed mature peptide encoded by BmKb1* (i.e., marmelittin) is boxed in *orange* and the premature stop codon is shown by “ξ” in *red*.

MeuFSPL-1 and MeuFSPL-2 are two *M. eupeus* svAMPs deduced from their nucleotide sequences (Table S2). The precursors are composed of a hydrophobic N-terminal signal peptide, an amphiphilic mature peptide of 18 residues followed by a charged amino acid-rich C-terminal propeptide (Figure 1). There is a five-residue (GKRRR) processing signal between the mature peptide and the propeptide, in which KRRR perfectly match the recognition motif of subtilisin/kexin-like proprotein convertases (PCs) ([R/K]-Xn-[R/K]↓: X, any amino acid; n, the number of spacer amino acid residues, usually 0, 2, 4, or 6 (here 2); [R/K], either an arginine or a lysine; and ↓, the site of cleavage) (Duckert et al., 2004) and the Gly acts as the amide nitrogen donor for amidation of the C-terminal Lys. Thus, the maturation of MeuFSPL-1 and MeuFSPL-2 may involve at least four enzymes. In addition to a signal peptidase to remove the signal peptide, a PC is needed to cut off the propeptide, leaving a product carrying a basic C-terminus that will be further removed by carboxypeptidase M, a protease specifically removing C-terminal basic residues (R or K) from protein precursors (Tan et al., 2003). Eventually, the remaining Gly-extended peptide will be converted into a des-Gly peptide amine by an α-amidating enzyme to create a C-terminally amidated mature peptide (Merkler, 1994; Figure 1). The reaction by carboxypeptidase M also occurs in many scorpion Na^+^ channel toxins to remove their C-terminal extra basic residues, such as -CH**R**, -CH**RR**, -CQ**R**, CT**R**, and CHS**R** (underlined and boldfaced) (Martin-Eauclaire et al., 1992; Zhu et al., 2012b).

MeuFSPL-1 differs from MeuFSPL-2 by one amino acid at site 15 (S15N) and interestingly, its mature peptide is chemically identical to the ortholog BmKb1 from *M. martensii* (Zeng et al., 2004) although they have five-amino acid differences in other regions (Figure 1). The presence of identical venom peptides among different scorpion species is really rare since there is only one such example reported so far, in which the toxin TsTX-Kβ from *Tityus serrulatus* is chemically identical to TstβKTx from *T. stigmurus* (Diego-García et al., 2008).

A database search revealed a great number of MeuFSPL-related peptides widely distributed in a diversity of scorpion species belonging to the family Buthidae (Figure 1). These svAMPs exhibited a detectable sequence similarity and a common C-terminally amidated modification to some frog skin-derived AMPs, such as PBN1 from *Phyllobates bicolor* (Vanhoye et al., 2003), PSN-14 from *P. azurea* (Thompson et al., 2007), and PLS-J7 from *P. jandaia* (Rates et al., 2011; Figure 1). This is the reason why we named these two peptides MeuFSPL-1 and MeuFSPL-2 (FSPL, Frog Skin Peptide-Like).

### Marmelittin, melittin-like peptide arose from FSPL svAMP

**Figure 2:**
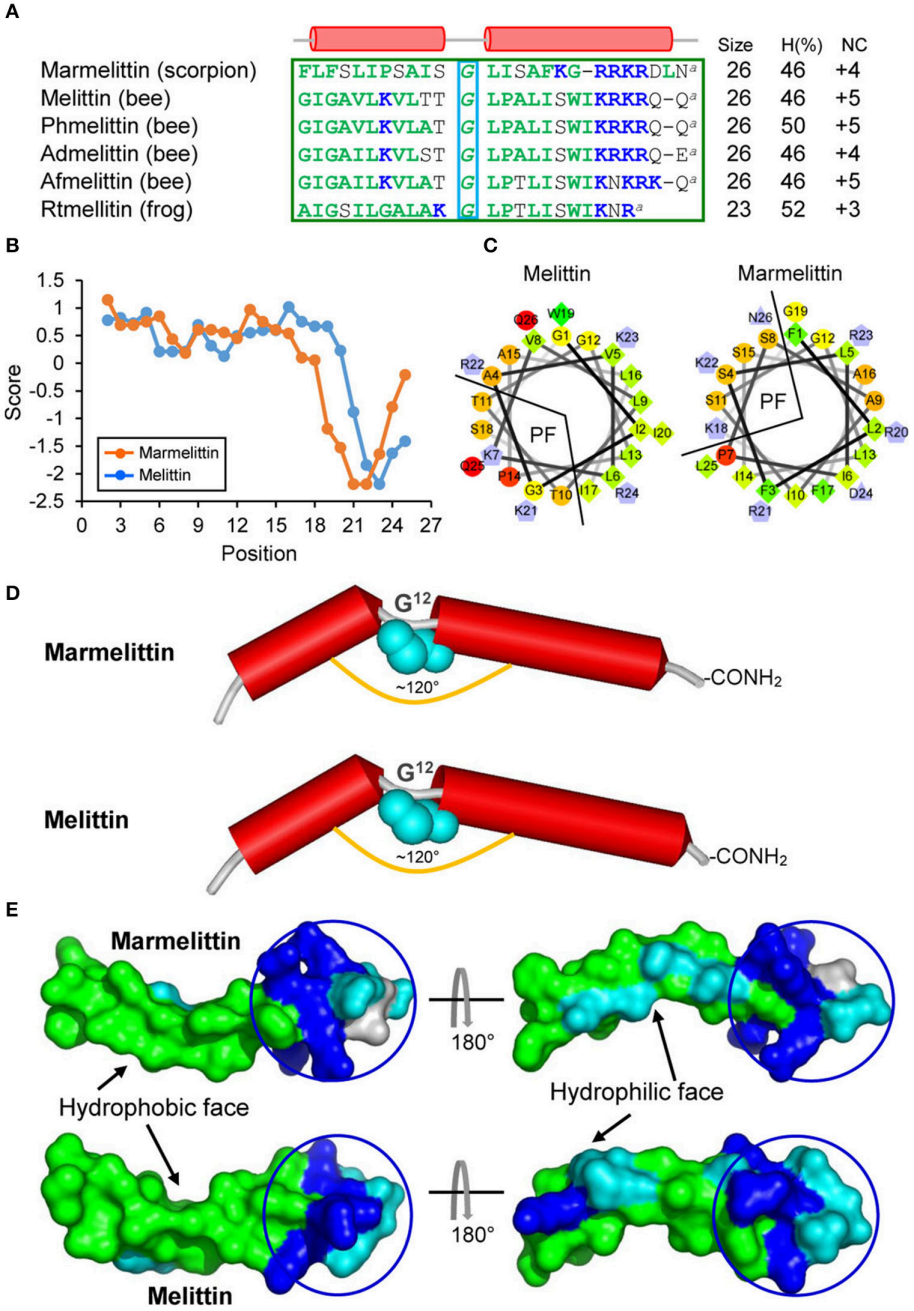
Marmelittin and Melittins. **(A)** Amino acid sequences. Hydrophobic and cationic residues are shown in *green* and *blue*, respectively. “^a^,” C-terminal amidation; α-helices, indicated by red cylinder, are extracted from the structural coordination of melittin (pdb 2MLT). The structural Gly is boxed in *cyan*. H (%), the percentage of hydrophobic amino acids. NC, net charge. The source of the sequences used here: Marmelittin from *Mesobuthus martensii* (AF159979), melittin from *Apis mellifera* (NP_001011607.1); Phmelittin from *Polistes hebraeus* (P59261); Admelittin from *Apis dorsata* (XP_006611828); Afmelittin from *Apis florea* (P01504); Rtmellitin from the frog *Rana tagoi* (BAF74741). (**B)** Hydropathy plots showing hydropathy scores for all the amino acids in the peptides. (**C)** Helical wheel projections of marmelittin and melittin. Hydrophilic, hydrophobic, negatively charged, and positively charged residues are presented as circles, diamonds, triangles, and pentagons, respectively. The most hydrophobic residue is shown in *green*, and the amount of *green* is decreasing proportionally to the hydrophobicity. The most hydrophilic (uncharged) residues are coded *red*, and the amount of *red* is decreasing proportionally to the hydrophilicity. Charged residues are shown in *light blue*. PF, polar face. (**D)** Structural similarity between marmelittin and melittin. The conserved Gly^12^ disrupting the helix is shown as cyan spheres. (**E)** Molecular surfaces of marmelittin and melittin with hydrophobic and hydrophilic regions indicated in *green* and *cyan*, respectively. The C-terminal charged/polar region is circled. The experimental structure of melittin (pdb 2MLT) was used as template to generate the structure of marmelittin. The method is detailed in Materials and Methods.

In our previous study, we reported a unique cDNA clone (414 bp) isolated from the cDNA library of the *M. martensii* venom gland, whose nucleotide sequence is nearly identical to that of *BmKb1* except a T to A mutation leading to a change from TAT encoding Tyr to a premature stop codon TAA. This gene (initially named BmKb1^*^) encodes a truncated precursor of 49 amino acids that lacks the major part of the propeptide (Zeng et al., 2004; Figure 1). Remarkably, the predicted mature peptide shares three commonalities to melittins (Raghuraman and Chattopadhyay, 2007): (1) *Precursor processing*. They both exist as Gly-extended peptides after the removal of signal peptides, which are further converted into a des-Gly peptide amine by an α-amidating enzyme; (2) *Sequence composition features*. They both consist of 26 amino acids with two distinct subdomains: a predominantly hydrophobic N-terminal domain (residues 1–20) and a hydrophilic and cationic C-terminal domain with a stretch of polybasic residues (residues 21–26; Figures 2A,B). This kind of detergent-like architecture is important for melittin to destroy bacterial membrane; *3*) *Structural features*. Firstly, our helical wheel projections reveal a similar amphipathic architecture in the N-terminal 18 amino acids of these two peptides, which is composed of a large hydrophobic face and a small polar face (Figure 2C). Secondly, Gly^12^, a structural residue in melittin, is also conserved in this peptide. It is known that this residue breaks the long helix of melittin, leading to the formation of a helix-bent-helix conformation with an angle of ~120° between two helices (Raghuraman and Chattopadhyay 2007). In our model structure, this new peptide folds into a kink-containing helical conformation (Figure 2D) with a similar molecular surface feature to that of melittin (Figure 2E). Although the truncated precursor retains a putative PC recognition motif (RRKR), further processing by a PC seems impossible because it lacks a C-terminal propeptide. This is further strengthened by the C-terminal sequence similarity between promarmelittin (RRKRDLNG) and promelittin (KRKRQQG). In melittin, only the glycine (underlined once) is removed for C-terminal amidation (Figure 2; Habermann, 1972). We proposed the name of marmelittin for this new melittin-related peptide.

**Figure 3:**
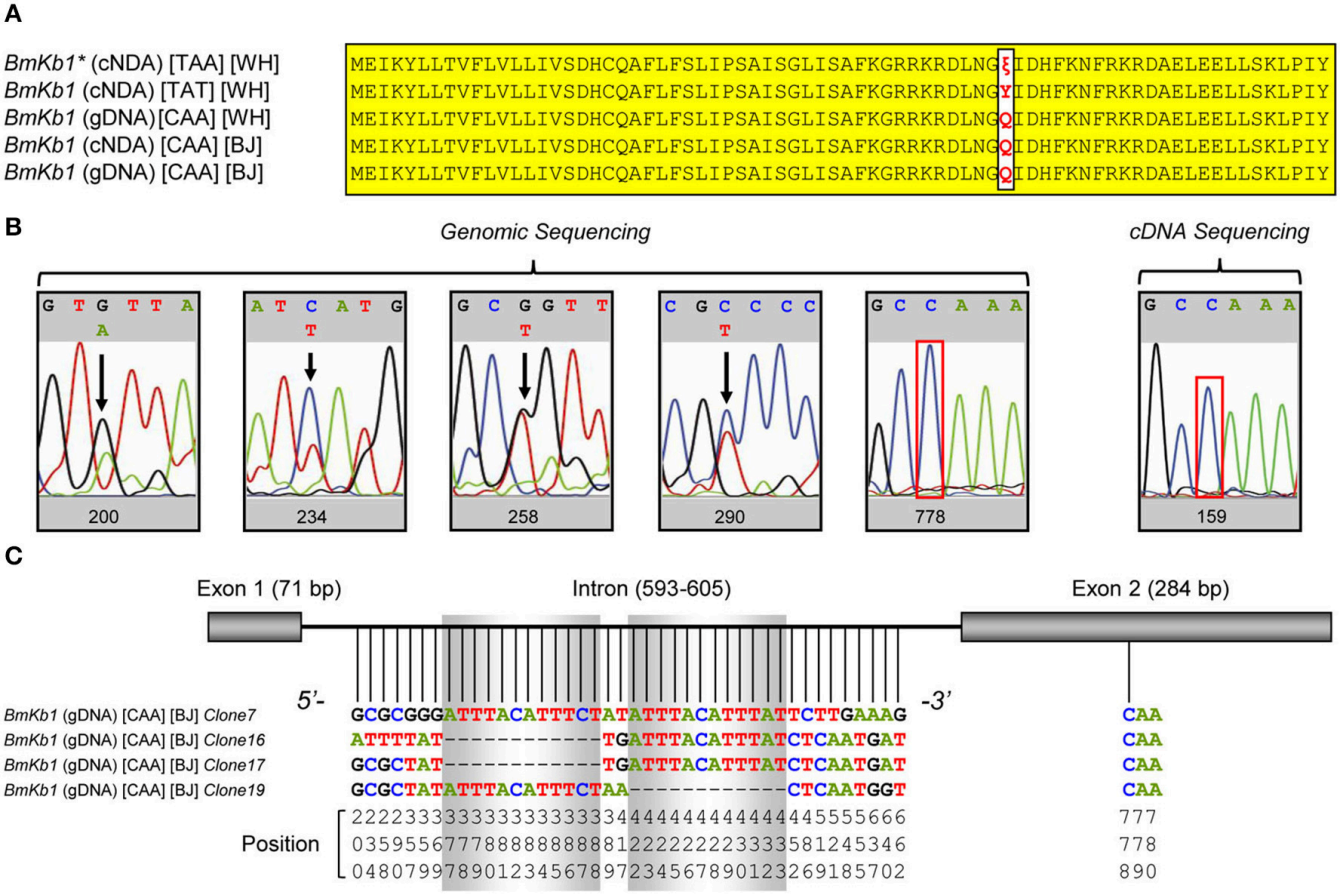
Polymorphism of *BmKb1*. **(A)** Precursor proteins encoded by *BmKb1* and its mutants. Variations at site 50 are shown in *red* and identical sites are shadowed in *yellow*. (**B)** Sequencing PCR products amplified from genomic DNA and cDNA for identifying polymorphic sites. **(C)** Sequencing different genomic clones identifying polymorphism in the intron region of *BmKb1*.

The single nucleotide substitution between *BmKb1* (cDNA) (TAT) and BmKb1^*^ (cDNA) (TAA) (Figure 3A) could arise from single nucleotide polymorphism (SNP) among individuals (Barreiro et al., 2008) or RNA editing altering specific genomic sequences (Gray, 2012). To distinguish between these two possibilities, we performed PCR sequencing of *BmKb1* amplified from the *M. martensii* gDNA or cDNA template. The results showed that despite the presence of some polymorphic sites in the intron region of the gene, no polymorphism occurred in its exon region, including both the gDNA and cDNA. The codon previously observed as TAT or TAA in the cDNA clones isolated from the Wuhan isolate of *M. martensii* became CAA in both gDNA and cDNA from the Beijing isolate (Figures 3A,B). In a previous study, Luo et al. also isolated a genomic clone with the codon CAA (61) (Figure 3A). Subsequent sequencing of multiple positive clones derived from ligation of the genomic PCR product into T-vector confirmed that SNPs and insertion/deletion (indel) mutations in the intron region (Figure 3C). PCR sequencing definitely ruled out the possibility of RNA editing. Alternatively, SNP resulted from the C → T substitution is a more plausible explanation for the origin of the premature codon. Given that among all the currently known SNPs, 2/3 result from C → T substitution, this C → T substitution should not be an occasional case. Our PCR sequencing of gDNA failed to detect a variation at the allele could be due to different mutation frequency between *M. martensii* isolates used here (Beijing vs. Wuhan in China). Obviously, CAA appears in most individuals whereas TAA or TAT exists in a minority of individuals.

Multiple mutations occurring at this codon (i.e., CAA, TAT, and TAA) (Zeng et al., 2004; Luo et al., 2005; this work) also excludes the possibility of an artifact generated during reverse transcription because even errors committed by reverse transcriptase (RT), they should occur at random rather than focusing on a single site (Ellefson et al., 2016).

### Revision of promeucin-18 processing

**Figure 4:**
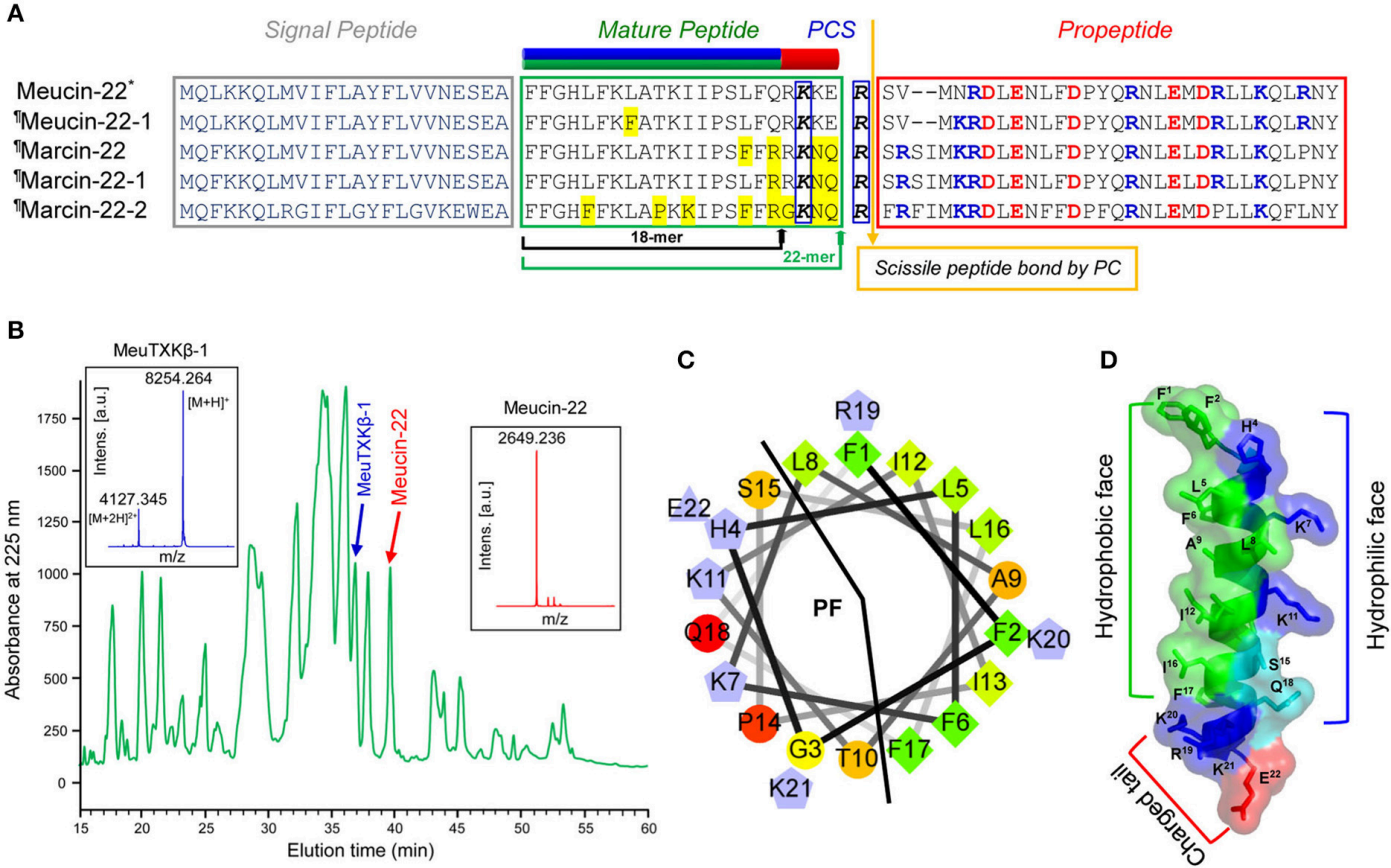
Meucin-22 and homologs. **(A)** Precursor amino acid sequences. The *green* arrow indicates a newly proposed cleavage site based on the presence of a putative proprotein convertase signal (PCS), which leads to a C-terminal extension of four residues relative to the initially proposed Meucin-18, indicated by a *black* arrow. In the mature region, the amino acids non-identical to Meucin-22 are shadowed in *yellow* and in the proregion, charged residues are colored (basic, *blue*; acidic, *red*). The secondary structures of Meucin-22 and its homologs comprising an N-terminal amphipathic domain (residues 1–18, *green* and *blue*) and a charged tail (residues 19–22, *red*) are showed at the top of the sequence alignment. **(B)** Isolation of native Meucin-22, along with MeuTXKβ-1 from the venom of *M. eupeus* by RP-HPLC. Inset, MALDI-TOF MS of Meucin-22 and MeuTXKβ-1. For the spectrum of the latter, there are two main peaks, corresponding to the singly and doubly protonated forms of this peptide. **(C)** Helical wheel projection of Meucin-22. Color codes of amino acids are identical to those of Figure 2C. **(D)** Ribbon representation of Meucin-22 backbone structure that was built based on the experimental structure of racemic Ala-(8,13,18) Magainin 2 (pdb 4MGP), an analog of frog skin AMP. Residues with different side-chain natures are indicated in different colors (*blue*, positively charged; *red*, negatively charged; *green*, hydrophobic; *cyan*, polar) on the background of a semitransparent surface. In the helix formed by the N-terminal 18 residues, all hydrophobic residues are located on one surface while cationic and hydrophilic residues on another surface.The figure was prepared with PyMOL (http://www.pymol.org).

Using a PCR strategy, we isolated four new cDNA clones encoding Meucin-18-related peptides: one from *M. eupeus* (named Meucin-22-1) and three from *M. martensii* (named Marcin-22, Marcin-22-1, and Marcin-22-2; Figure 4). Based on the criterion that the bioactive peptide moiety lies upstream or downstream of a propeptide separated by a single or dibasic motif (Lys/Arg-Arg), we initially predicted Arg^42^ as the processing signal for the maturation of Promeucin-18 (Gao et al., 2009). However, when analyzing these newly cloned precursor sequences, we found that this basic residue was substituted by a glycine in Marcin-22-2, promoting us to search for other more likely processing signal. As shown in Figure 4, there is a common recognition signal of PCs conserved across all the members (^43^**K**KE**R**^46^ in *M. eupeus* and ^43^**K**NQ**R**^46^ in *M. martensii*; Figure 4A). Thus, it is reasonable to assume that the propeptides of these precursors are firstly removed by PC cleavage after Arg^46^ and then this arginine is cut off by carboxypeptidase M, leaving a four-amino acid extension in their C-termini relative to Meucin-18 and thus named Meucin-22 and Marcin-22. This revision is verified by the purification of a native peptide from the venom of *M. eupeus* (Figure 4B), which had an experimental molecular weight (MW) of 2649.236 Da determined by MALDI-TOF and an N-terminal sequence of FFGHLFKLAT determined by Edman degradation, both perfectly matching Meucin-22. In this initial purification, Meucin-22 was co-eluted with chymotrypsin-like proteases and thus repurification was done by modifying the elution condition prior to Edman degradation (Figure S1, provided as Supplementary Materials). Structurally, Meucin-22 is divided into two distinct subdomains comprising the N-terminal amphipathic domain corresponding to Meucin-18 and the C-terminally-extended charged tail (Figures 4C,D).

### Secondary structures of svcAMPs

**Figure 5:**
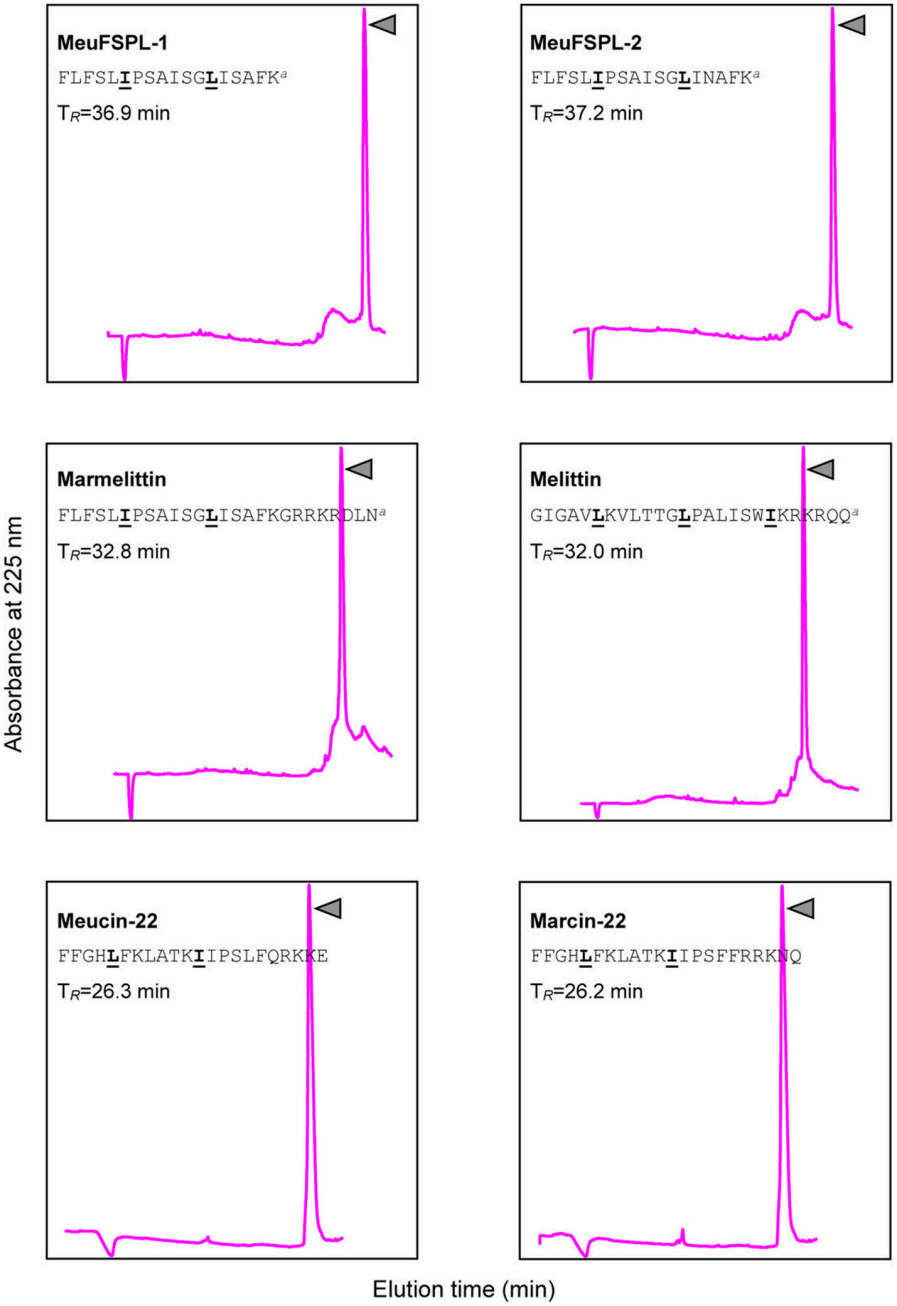
Purification of synthetic svAMPs by RP-HPLC. For each peptide, their sequences and retention times (T_*R*_) are shown. Pure peptides indicated by triangle were collected for structural and funtional studies. The leucine/isoleucine residues presumably forming a short leucine zipper-like motif are marked in *bold* and underlined once.

**Figure 6:**
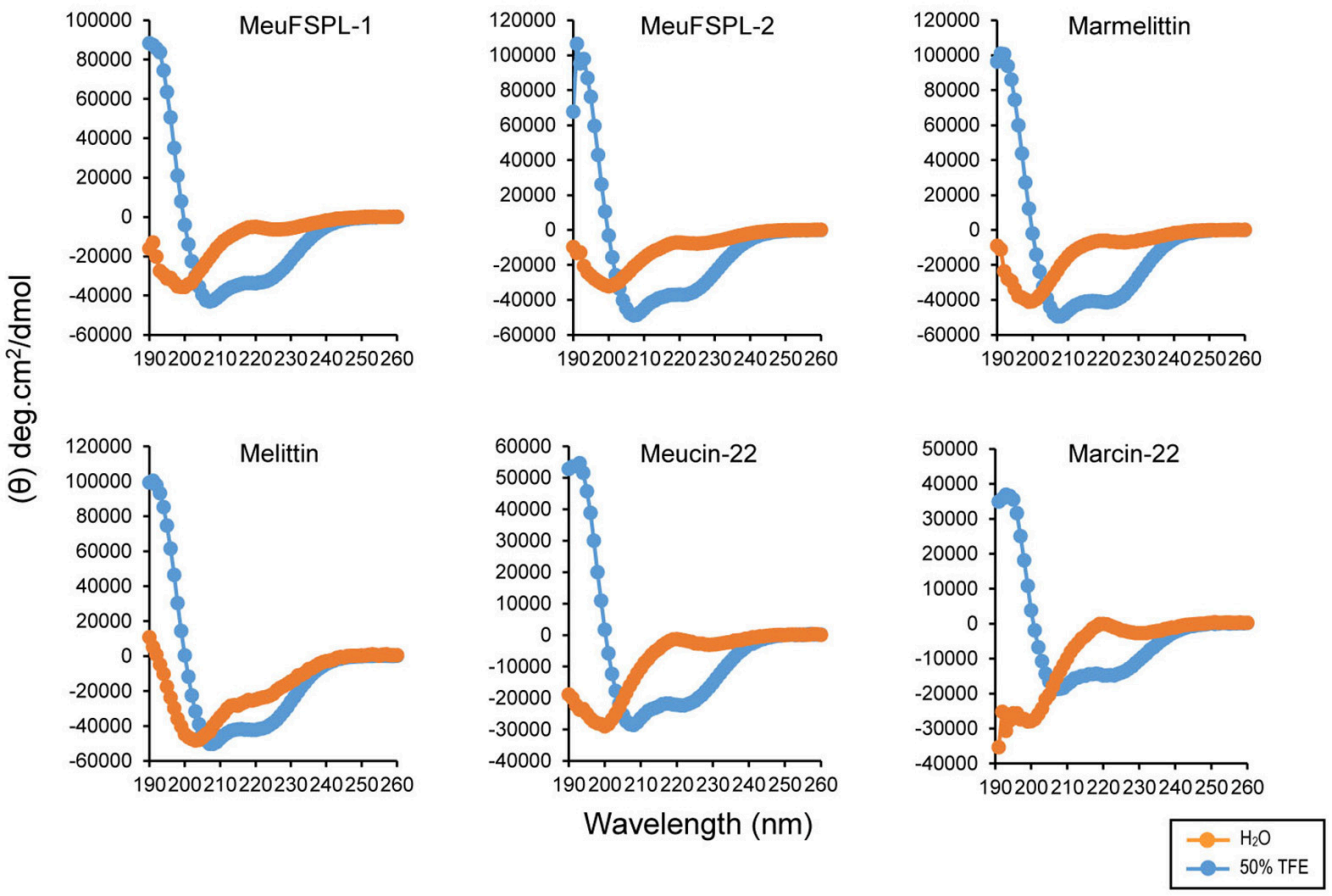
Circular dichroism spectra of svAMPs recorded in H_2_O and 50% TFE. Peptide concentrations used here were 0.1–0.15 mg/mL.

The purification of chemically synthesized svAMPs by RP-HPLC is shown in Figure 5. Using circular dichroism (CD) spectroscopy, we analyzed the structural features of these svAMPs, including MeuFSPL-1, MeuFSPL-2, marmelittin, Meucin-22, and Marcin-22. Melittin, a well-known bee venom-derived α-helical AMP (Raghuraman and Chattopadhyay, 2007) was measured in parallel for comparative purposes. The results showed that all the svAMPs adopt a random coli in H_2_O, as identified by their very low ellipticity above 210 nm with strong negative minima at 200 nm and weak negative minima around 225–230 nm (Figure 6). However, similar to melittin, these svAMPs significantly changed their CD spectra in a membrane-mimicking environment (50% TFE), where the negative minima appeared at 208 and 222 nm and a positive band around 190 nm (Figure 6), typicaly indicative of α-helical conformation.

### Antimicrobial properties of svAMPs

Using a diversity of microbial strains, we evaluated the antimicrobial spectra of the svAMPs described here. For the svcAMPs, their antibacterial properties against the Gram-positive bacteria can be summarized as follows: (1) They displayed a broad spectrum of activity against a variety of Gram-positive bacteria, such as *B. cereus, B. megaterium, B. subtilis, M. luteus, S. aureus, S. epidermidis, S. warneri, S. griseus, S. scabiei, S. mutans, S. salivarius*, and *S. sanguinis* (Table 1). (2) The majority of them possessed a high bactericidal potency with lethal concentrations (C_*L*_) < 10 μM, and particularly for several peptides (e.g., Meucin-22, Marcin-22 and Marmelittin), their C_*L*_ over some Gram-positive bacteria were even <2 μM (0.18–1.85 μM). (3) Similar to Meucin-13, MeuFSPL-1 and MeuFSPL-2 showed a relatively weak activity than the longer α-helical svAMPs. (4) In all cases, marmelittin was more active than melittin.

**Table 1:** Lethal concentrations (C_*L*_, μM) of svAMPs against Gram-positive bacteria.

**Bacteria**	**MUC-22**	**MAC-22**	**MUC-18**	**MUC-13**	**MeuFSPL-1**	**MeuFSPL-2**	**MarMEL**	**Melittin**	**MeuTXKβ1**
*BC* CGMCC 1.1846	N.D.	N.D.	N.D.	N.D.	15.33	19.57	6.69	24.74	N.A.
*BM* CGMCC 1.0459	3.72	1.35	0.25*	0.25*	2.10	2.67	2.86	11.6	0.29
*BS* CGMCC 1.2428	1.86	2.11	2.11	6.24	4.74	4.01	3.13	9.2	0.54
*ML* CGMCC 1.0290	2.71	2.58	0.60*	2.90*	2.26	1.50	1.85	7.5	0.33
*MSSA* CGMCC 1.89	1.35	1.35	0.87	10.24	5.27	3.14	4.87	7.9	N.A.
*PSSE* P1111	1.61	1.35	0.87	10.24	7.36	4.77	4.24	13.4	N.A.
*MRCNS* P1369	2.21	1.35	4.89	33.80	8.48	3.88	3.01	9.3	2.15
*MRSA* P1374	0.87	1.35	3.35	N.A.	6.16	3.61	2.60	11.6	10.41
*PRSA* P1383	0.87	2.11	3.72	N.A.	2.81	3.52	2.35	9.1	0.88
*MRSA* P1386	1.88	1.35	1.35	N.A.	6.24	5.13	8.11	9.3	N.A.
*PRSE* P1389	0.56	3.72	3.35	24.06	7.36	5.13	3.12	9.1	2.15
*SA* J685	2.66	2.39	8.71	10.16	14.72	5.36	8.41	13.70	N.A.
*SA* J698	2.66	2.39	5.16	7.54	4.80	4.11	6.24	12.08	N.A.
*SA* J700	2.66	2.39	6.86	7.54	10.92	8.44	10.86	12.08	N.A.
*SA* J706	N.D.	N.D.	4.86	5.67	10.92	7.04	8.41	16.41	16.90
*SA* J708	N.D.	N.D.	6.86	7.54	10.92	6.21	4.69	7.80	N.A.
*SA* J710	N.D.	N.D.	5.16	7.10	10.92	7.04	8.41	8.98	N.A.
*SSAN* ATCC 1.2497	3.37	9.12	13.72	6.24	5.20	2.70	4.70	16.41	14.26
*SSAL* ATCC 1.2498	4.22	4.22	9.24	7.54	4.80	5.36	4.70	18.20	17.68
*SM* ATCC 1.2499	2.39	4.22	9.32	10.24	4.22	5.23	2.85	9.37	13.76
*SW* ATCC 1.2824	3.76	4.45	11.69	6.24	2.81	8.65	0.18	1.37	24.06
*SG* NBRC 13350	N.D.	N.D.	31.40	53.31	6.81	N.A.	5.19	28.79	33.80
*SSCA* CGMCC 4.1765	N.D.	N.D.	4.45	15.07	1.77	7.75	1.56	15.93	N.A.

MUC, Meucin; MAC, Marcin; MarMEL, Marmelittin; BC, Bacillus cereus; BM, Bacillus megaterium; BS, Bacillus subtilis; ML, Micrococcus luteus; MRCNS, methicillin-resistant coagulase-negative Staphylococcus; MRSA, methicillin-resistant Staphylococcus aureus; MSSA, methicillin-sensitive Staphylococcus aureus; PRSA, penicillin-resistant Staphylococcus aureus; PRSE, penicillin-resistant Staphylococcus epidermidis; PSSE, penicillin-sensitive Staphylococcus epidermidis; SA, Staphylococcus aureus; SW, Staphylococcus warneri; SM, Streptococcus mutans; SSAL, Streptococcus salivarius; SSAN, Streptococcus sanguinis; SG, Streptomyces griseus; SSCA, Streptomyces scabiei. “N.A.” means “no activity” at 0.8 nmol peptide each well in our inhibition zone assay. “N.D.” means “not determined”. “^*^” represents data that were previously published (Gao et al., 2009).

Despite these peptides exhibited a lower activity on the Gram-negative bacteria than they did on the Gram-positive bacteria, we observed an overall similar trend (Table 2). For example, Meucin-22, Marcin-22, marmelittin, and Meucin-18 had a wide spectrum of activity against the Gram-negative bacteria, including *A. faecalis, E. coli, P. aeruginosa, P. solanacearum, S. enterica, S. marcescens*, and *S. maltophilia* (Table 2), whereas Meucin-13, MeuFSPL-1 and MeuFSPL-2 showed a relatively narrow anti-Gram-negative bacterial spectrum. Again, marmelittin showed more potency on Gram-negative bacteria than melittin did (roughly 2–3-folds). In particular, marmelittin other than melittin showed some weak but detecable activity on a series of *P. aeruginosa* strains isolated from animals and humans (Table 2). Comparison of the functional data between the long and short forms (e.g., Meucin-22 and Meucin-18; marmelittin and MeuFSPL-1) revealed that the former exhibited stronger activity on both Gram-positive and negative bacteria than did the latter (Tables 1, 2), highlighting the functional importance of the C-terminal extension in these peptides. In our assays, only three svAMPs (MeuFSPL-1, MeuFSPL-2, and marmelittin) were characterized to have a comparatively wide-spectrum antifungal activity against the filamentous fungi and a series of yeasts (Table 3).

**Table 2:** Lethal concentrations (C_*L*_, μM) of svAMPs against Gram-negtive bacteria.

**Bacteria**	**MUC-22**	**MAC-22**	**MUC-18**	**MUC-13**	**MeuFSPL-1**	**MeuFSPL-2**	**MarMEL**	**Melittin**	**MeuTXKβ1**
*AF* CGMCC 1.1837	11.65	11.13	18.64	N.A.	N.A.	N.A.	N.A.	N.A.	N.A.
*EC* ATCC 25922	7.95	10.69	2.40*	7.90*	12.50	12.16	6.41	14.4	N.A.
*EC* DH5α	N.D.	N.D.	N.D.	N.D.	N.A.	N.A.	5.48	18.20	N.D.
*EC* JM109	N.D.	N.D.	N.D.	N.D.	N.A.	N.A.	5.55	23.53	N.D.
*EC* TOP10	N.D.	N.D.	N.D.	N.D.	9.94	11.58	4.10	13.80	N.D.
*EC* Am. J16c	7.49	15.15	22.72	N.A.	43.66	N.A.	7.75	24.54	8.76
*EC* Am. J23a	7.36	12.70	22.72	N.A.	25.00	37.43	4.69	18.20	15.91
*EC* CIP. J14b	11.65	12.03	22.72	N.A.	N.A.	37.43	8.41	18.20	12.03
*EC* D. G2b	9.93	12.95	37.28	N.A.	25.00	21.42	6.24	18.20	29.77
*EC* D. J45b	7.49	6.78	22.72	N.A.	N.A.	N.A.	6.24	24.54	16.90
*PA* O1	3.79	12.03	41.58	N.A.	N.A.	N.A.	N.A.	N.A.	29.77
*PA* 14	4.06	6.78	31.40	N.A.	N.A.	N.A.	N.A.	N.A.	39.44
*PA* FRD1	16.86	18.75	N.A.	N.A.	N.A.	N.A.	N.A.	N.A.	N.A.
*PA* 374	16.86	18.75	N.A.	N.A.	N.A.	N.A.	N.A.	N.A.	39.44
*PA* 11082603	6.10	14.35	N.A.	N.A.	N.A.	N.A.	8.41	N.A.	29.77
*PA* 11082616	10.02	19.08	31.40	N.A.	N.A.	N.A.	8.41	N.A.	N.A.
*PA* 11092304	10.02	24.82	31.40	N.A.	N.A.	N.A.	24.95	N.A.	39.44
*PA* 11092618	6.10	19.08	31.40	N.A.	N.A.	N.A.	8.41	N.A.	39.44
*PA* DH	9.93	11.13	N.A.	N.A.	N.A.	N.A.	14.29	N.A.	39.44
*PA* QT1	6.10	10.98	18.49	N.A.	N.A.	N.A.	14.29	N.A.	25.98
*PS*	21.61	22.08	N.A.	N.A.	N.A.	N.A.	N.A.	N.A.	N.A.
*SE* ATCC 14028	14.73	16.37	35.64	N.A.	N.A.	N.A.	1.61	24.74	29.77
*SMAR* ATCC 14041	58.93	N.A.	N.A.	N.A.	N.A.	N.A.	N.A.	N.A.	N.A.
*SMAL* CGMCC 1.1788	3.79	6.78	13.72	4.89	1.13	3.52	4.14	18.72	52.01

AF, Alcaligenes faecalis; EC, Escherichia coli; PA, Pseudomonas aeruginosa; PS, Pseudomonas solanacearum; SE, Salmonella enterica; SMAR, Serratia marcescens; SMAL, Stenotrophomonas maltophilia. “N.A.” means “no activity” at 0.8 nmol peptide each well. “N.D.” means “not determined”. “^*^” represents data that were previously published (Gao et al., 2009).

**Table 3:** Lethal concentrations (C_*L*_, μM) of svAMPs.

**Fungi**	**MeuFSPL-1**	**MeuFSPL-2**	**MarMEL**	**MeuTXKβ1**
**FILAMENTOUS FUNGI**				
*ANID* A28	14.84	N.A.	7.14	N.D.
*ANID* Rcho15	N.A.	N.A.	5.65	N.D.
*GC* CCTCC AY 93038	17.70	24.94	6.34	N.D.
**YEASTS**				
*CA* JX1195	N.A.	N.A.	5.65	N.A.
*CA* JX1009	15.55	11.58	6.49	N.A.
*CA* JX1016	28.38	23.35	14.29	N.A.
*CA* JX1017	11.72	11.10	4.07	N.A.
*CA* 4247	15.28	7.02	7.04	N.A.
*CA* 4257	14.64	12.72	6.84	N.A.
*CA* 4259	14.80	12.61	5.17	N.A.
*CA* 4277	6.25	7.06	7.04	N.A.
*CA* SZ56	21.82	14.80	8.49	N.A.
*CA* SZ72	21.82	12.72	6.70	N.A.
*PP* X33	8.21	N.A.	3.57	N.A.
*SC* CCTCC AY 92003	25.00	11.62	7.72	N.A.

ANID, Aspergillus nidulans; GC, Geotrichum candidum; CA, Candida albicans; PP, Pichia pastoris; SC, Saccharomyces cerevisiae. “N.A.” means “no activity” at 0.8 nmol peptide each well. “N.D.” means “not determined.”

### Hemolysis and pain-induction by svcAMPs

**Figure 7:**
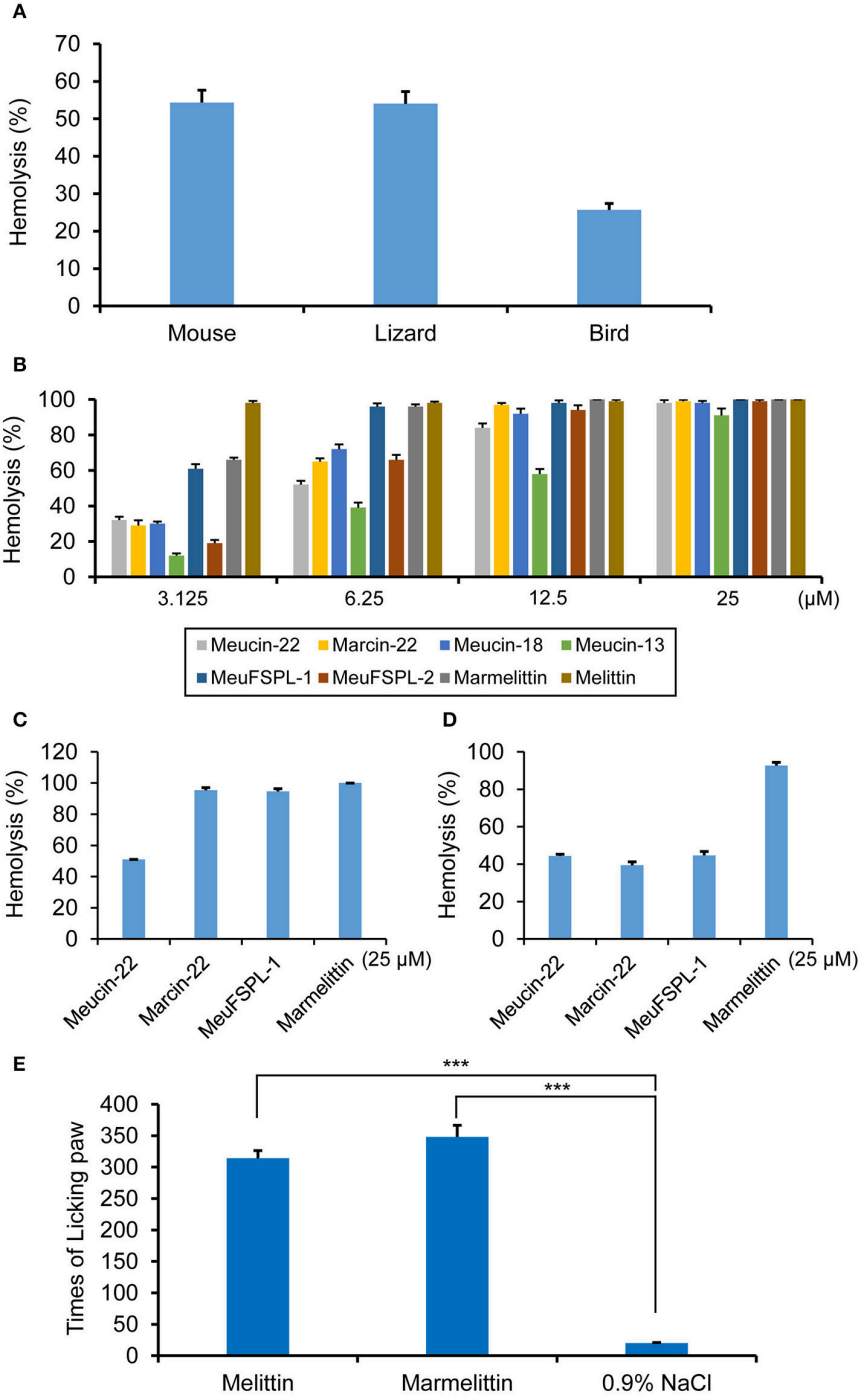
Hemolysis and pain induction by svAMPs. **(A)** Hemolytic activity of the crude venom of *M. eupeus* on mouse, lizard and bird erythrocytes. **(B)** Hemolysis of mouse erythrocytes by the svAMPs as a function of peptide concentration. Melittin was used as positive control. Hemolysis of lizard **(C)** and bird **(D)** erythrocytes by the peptides at 25 μM. Erythrocytes were suspended in PBS buffer and incubated with different concentrations of peptide for 30 min at 37°C. The absorbance of the supernatant was recorded at 570 nm. Controls for 0 and 100% hemolysis were determined by PBS buffer and 1% Triton X-100, respectively. Assays were repeated in triplicate and percentages of hemolysis are expressed as mean ± S.D. **(E)** Times of licking paw of mice induced by melittin and marmelittin, recorded between 5 and 30 min post-injection of peptides (*n* = 3). 0.9% NaCl was used as control. Scale bars represent means from three independent experiments; error bars, standard deviations (SDs). ***P < 0.001 (compared with the control without treatment by the peptides).

Previous literatures have reported the hemolytic activity of some svcAMPs on mammalian blood cells (Corzo et al., 2001; Gao et al., 2009; Harrison et al., 2016). This activity has been considered as a defensive strategy in repelling small mammals. To provide more evidence to support this speculation, we collected blood cells from three most commonly vertebrates preying on scorpions (mice, lizards, and birds; Polis, 1990) to assess hemolytic activity of *M. eupeus* crude venom and single svcAMPs. As shown in Figure 7A, the venom diluted by 0.9% NaCl led to > 50% of hemolysis on both mouse and lizard blood cells and 25.7 ± 1.7% of hemolysis on the bird blood cells (Figure 7A). For single peptide components, at a peptide concentration of 3.125 μM, all the svcAMPs exhibited different extents of hemolysis on mouse blood cells, in which MeuFSPL-1 and marmelittin showed more potent hemolysis (61 ± 2.5% and 66 ± 1.2%, respectively) than others. At this concentration, melittin led to complete hemolysis. With the increase of the peptide concentraiton to 6.25 μM, a large majority of svcAMPs had hemolytic activity exceeding 50% whereas MeuFSPL-1 and marmelittin both showed elevated hemolysis to nearly 100%. When the peptide concentration reached 12.5 μM, nearly all the peptides except Meucin-13 led to complete hemolysis. 25 μM was required for Meucin-13 to obtain the same effect. Taken together, the hemolytic activity of these peptides can be ranked as follows: melittin > MeuFSPL-1 = marmelittin > Meucin-22 = Marcin-22 = Meucin-18 = MeuFSPL-2 > Meucin-13 (Figure 7B). At 25 μM some of them led to complete hemolysis of lizard blood cells (Figure 7C). Except marmelittin that exhibited nearly complete hemolysis of the bird's blood cells at 25 μM, other peptides resulted in partial hemolysis (Figure 7D). The observation that MeuFSPL-1 and MeuFSPL-2 differ by only one substitution but their hemolysis significantly differ strengthens the necessity of investigating each scorpion venom component even if they differ by only one amino acid. The hemolytic activity of the svcAMPs could be related to their sequences that all possess a short leucine zipper-like motif (Figure S2). This motif, defined by every seventh amino acid as leucine/isoleucine, has been identified as a crucial structural element conferring the hemolytic activity of melittin (Asthana et al., 2004; Hayouka et al., 2013).

Because the highly hemolytic melittin has been identified as the major pain-producing substance of bee venom (Chen et al., 2016), we wanted to know whether marmelittin has a similar effect. To do this, we injected the peptide into the right hinder sole of mice to observe their pain response. A frequent paw-licking behavior appeared 5 min post-injection. The paw-licking times recorded were 348 ± 18 (*n* = 3), comparable with 315 ± 12 times recorded from melittin (*n* = 3; Figure 7E). For the mice injected with the saline, we did not observe an obvious paw-licking behavior. The time for appearance of paw-licking behavior indicates that this class of peptides specifically induce inflammatory pain likely via simulating release of inflammatory mediators (Kidd and Urban, 2001), instead of imposing a direct chemical stimulation of nociceptors to induce neurogenic pain (Bowsher, 1991).

### Insecticidal activity of svAMPs

**Figure 8:**
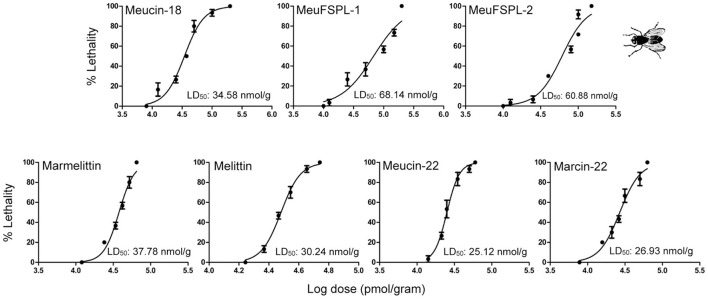
Dose-response curves resulting from injection of peptides into housefly adults. Data points are the mean ± SD of three experiments. LD_50_ of each svAMP was calculated from the dose-response curves. Melittin was used as control.

Some α-helical AMPs derived from spider venoms possess insecticidal activity (Kuhn-Nentwig et al., 2011), but little data are reported on this activity in svcAMPs. Prior to this work, only Im-1, a svAMP from the venom of *Isometrus maculatus*, was found to have paralysis to *Acheta domesticus* but no lethality even at a relatively high dose (40 nmol/g; Miyashita et al., 2010). For this purpose, we evaluated the insecticidal activity of these svcAMPs (Meucin-18, MeuFSPL-1, MeuFSPL-2, marmelittin, Meucin-22, and Marcin-22) on housefly adults. The results show that unlike Im-1, all these peptides were lethal to this pest species in a concentration-dependent manner (Figure 8). Based on the dose-response curves, we calculated their half maximal lethal dose (LD_50_) (Figure 8), from which we can see that the majority of them exhibited a similar insecticidal potency with a LD_50_ ranging from 25.12 to 37.78 nmol/g, except for MeuFSPL-1 and MeuFSPL-2 that had a 2-fold lower activity. Compared with scorpion neurotoxins affecting Na^+^ channels (Zhu et al., 2016), these svcAMPs overall showed weaker insect toxicity.

### MeuTXKβ1: a two-domain peptide with wide-spectrum antibacterial activity

**Figure 9:**
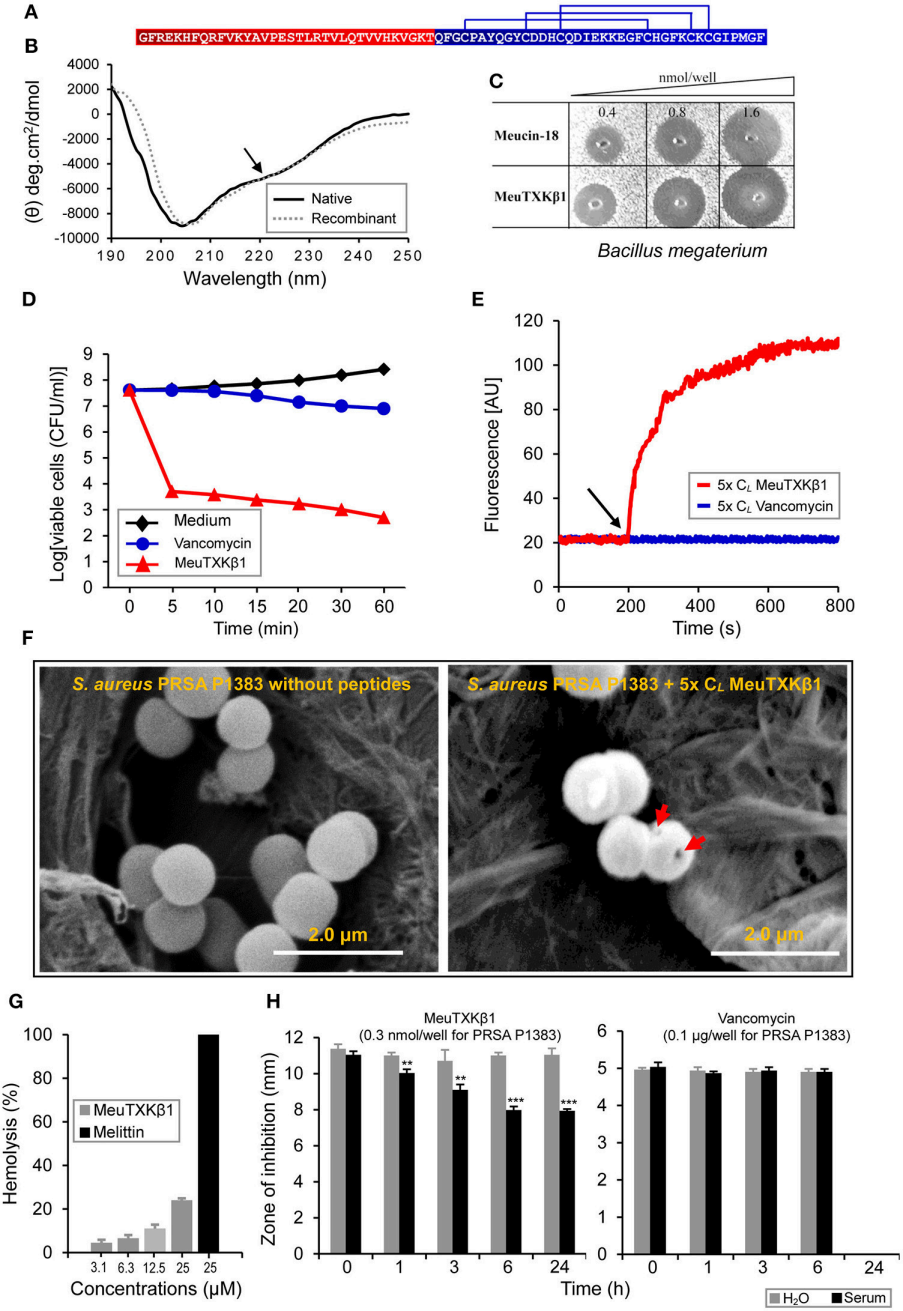
Sructural and functional features of MeuTXKβ1. **(A)** The sequence of MeuTxKβ1. Two distinct domains are highlighted in color and three disulfide bridges are also shown here. **(B)** Comparison of CD spectra between native and recombinant MeuTxKβ1 (Zhu et al., 2010). The recombinant product was a mixture of the full-length peptide and a truncated peptide with the N-terminal three residues (Gly-Phe-Arg) removed (Zhu et al., 2010). Peptide concentrations used here were 0.3 mg/ml. A minor negative band around 222 nm in the native peptide is indicated by an arrow. **(C)** Comparison of the anti-*B. megaterium* activity between MeuTXKβ1 and Meucin-18. **(D)** Killing kinetics of MeuTXKβ1. The *S. aureus* PRSA P1383 bacteria were treated with peptide solutions (specified in the figure, at 5x C_*L*_) for 5 to 60 min and survivors were plated. **(E)**. Membrane permeation ability of MeuTXKβ1 on *S. aureus* PRSA P1383 at 5x C_*L*_. In **(D,E)**, vancomycin was parallelly evaluated (5x C_*L*_ for P1383 = 20 μg/ml) for comparison purpose. **(F)** Scanning electron microscopic observation of MeuTXKβ1-induced bacterial deformation. **(G)** Hemolysis of MeuTXKβ1 on mouse erythrocytes. Melittin was used as control. **(H)** The stability of MeuTXKβ1. Peptides were incubated in H_2_O or mouse serum for the indicated times and then added to bacterial plates containing *S. aureus* PRSA P1383. Diameters of inhibition zone were recorded after incubation at 37°C overnight. In **(G,H)**, the data are presented as mean ± SD from three independent experiments. **P < 0.01; ***P < 0.001 (compared with the control without incubation in H_2_O or the serum).

MeuTXKβ1 is a svntAMP isolated from the venom of *M. eupeus* with some weak K^+^ channel blocking activity (Zhu et al., 2010). This two-domain peptide is composed of an N-terminal α-helical domain and a C-terminal defensin domain (Figure 9A). Data currently available on its structure and function was obtained by using recombinant peptide that was actually a mixture of the full-length molecule and a truncated peptide with three N-terminal residues removed (Zhu et al., 2010). The truncation appears to be responsible for its α-helical content decrease since in our published structure model, these removed residues adopt an α-helical conformation. To verify this, we isolated its native form from the *M. eupeus* venom (Figure 4B) and measured its CD spectra for comparison with its recombinant form. As shown in Figure 9B, the native peptide had a weak negative minimum at 222 nm (Figure 9B) that was lacking in the recombinant peptide (Zhu et al., 2010), suggesting that the former possessed a slightly higher α-helical content. In comparison with the recombinant peptide that only showed a marginal activity on *Stenotrophomonas* sp. strain YC-1 and even no effect on *Bacillus megaterium* (Zhu et al., 2010), native MeuTXKβ1 isolated here exhibited rather strong bactericidal effect on *B. megaterium* (Figure 9C), supporting the functional importance of the three N-terminal residues. Especially, it was capable of killing several Gram-positive clinical isolates of *Staphylococcus*, such as MRCNS P1369, PRSA P1383, and PRSE P1389, at low lethal concentrations (0.88–2.15 μM; Table 1). Moreover, native MeuTXKβ1 showed a wide spectrum of activity against the Gram-negative bacteria, such as *A. faecalis, P. aeruginosa, P. solanacearum, S. enterica, S. marcescens, S. maltophilia*, and some strains of *E. coli* (Table 2). It is a strict antibacterial peptide without activity on fungi used in this study (Table S3).

The killing curve of MeuTXKβ1 on *S. aureus* PRSA P1383 is shown in Figure 9D. This peptide led to a significant reduction in the number of CFUs/ml within 5 min and nearly 99.9% of bacteria killed within 60 min (Figure 9D). In parallel, we also measured the killing kinetics of vancomycin (Figure 9D), which showed a slower killing rate than MeuTXKβ1. This difference seems to be associated with their differential action modes (cell wall synthesis inhibition vs. rapid membrane disruption). The ability of MeuTXKβ1 in disrupting the membrane integrity of bacteria (*S. aureus* PRSA P1383) was studied using a fluorescent dye (propidium iodide, PI) that binds to DNA. When the peptides at 5x C_*L*_ were added to the bacterial cells pre-incubated with PI, the fluorescence rapidly increased due to the formation of PI-DNA complex (Figure 9E), indicative of membrane damage resulting in DNA release. In accord with this observation, scanning electron microscopy (SEM) showed that MeuTXKβ1 at 5x C_*L*_ led to visible damage in the bacterial surface structure of *S. aureus* PRSA P1383 (Figure 9F). Different from the highly hymolytic svcAMPs (Figure 7), MeuTXKβ1 only showed very weak hemolysis (Figure 9G), consistent with the absence of the short leucine zipper-like motif in its N-terminal helical region (Zhu et al., 2010).

Aside from low hemolysis, MeuTXKβ1 exhibited more serum stability when compared with the svcAMPs (Figure 9H). We found that except for MeuFSPL-2, all other svcAMPs and Melittin showed stablity after 24 h of incubation in H_2_O but only little or no activity remained after 24 h of incubation in mouse serum (Figure S3). Among these peptides, MeuFSPL-2 was the most unstable because its activity nearly disappeared by H_2_O or entirely lost by serum after only 4 h of treatment. In comparison with these svcAMPs, the activity of MeuTXKβ1 substantially remained even after treated by mouse serum for 24 h and the activity completely remained after treated by H_2_O for the same time (Figure 9H). The stability of this peptide could attribute to its three intramolecular disulfide bridges providing structural protection from protease degradation. In H_2_O or the serum, vancomycin remained its full activity after treated 6 h but the activity completely lose after 24 h (Figure 9H).

## Discussion

### Convergent evolution between svAMPs and frog skin-derived AMPs

In our prior work, we have observed sequence similarity and C-terminally amidated modification commonality between Meucin-13/Meucin-18 and related frog skin AMPs (Gao et al., 2009). Again, such similarity and commonality are found to exist between the short svAMPs reported here and other frog skin AMPs (Figure 1). In these examples, the similar mature sequences are very short and processed from different precursor organizations. These observations provide no support for their evolutionary link. Alternatively, evolutionary convergence could be a plausible explanation, in which similar selection pressure might promote their origin in distantly related species. Because some scorpion species often use to spray venom on their own body surfaces to clean them from saprophytic microbes (Torres-Larios et al., 2000), extensive convergence in multiple types of AMPs from scorpion venoms and frog skins suggests that these two evolutionarily distant animals convergently developed a similar strategy to combat a skin-borne bacterial infection, and that short-chain α-helical AMPs could be particularly effective in killing animal skin-borne microorganisms. Indeed, convergent evolution was also observed in the frog skin toxin caerulein from remote species (*Xenopus* and *Litoria*; Roelants et al., 2010) and melittin-related peptides from bees and frogs (Conlon et al., 2003).

### Antimicrobial immune function of svAMPs

In scorpions, liner svcAMPs are the most potent components of their venoms, which is remarkably different from the structurally compact scorpion hemolymph-derived AMPs. The latter are usually stabilized by two or three disulfide bridges (e.g., Androctonin, Buthinin, and Defensin from *Androctonus australis*, and Cll-dlp from *Centruroides limpidus* (Ehret-Sabatier et al., 1996; Rodríguez de la Vega et al., 2004), demonstrating that scorpions use two sets of independent AMPs in their hemolymph and venoms. Our studies reveal that the *M. eupeus* venom contains multiple svAMPs with different folds, molecular sizes and microbial selectivity. Overall, the svAMPs from *M. eupeus* showed more potent activity on Gram-positive bacteria than on Gram-negative bacteria. This is obviously different from venoms from scorpions *Leiurus abdullahbayrami, Parabuthus schlechteri*, and *Opistophthalmus carinatus*, which are more active on Gram-negative bacteria (Moerman et al., 2002; Erdeş et al., 2014). This difference could be a reflection of ecological adaptation of scorpions to different micobial species in their habitats, strengthening the roles of these peptides in scorpion immunity. These membrane-active svcAMPs (Gao et al., 2009) have molecular sizes ranging from 13 to 26. Importantly, the longer peptides are in possession of stronger potency, consistent with their different membrane-targeting mechanisms. It is known that AMPs attach to and insert into bacterial membrane bilayers to form pores by “barrel-stave,” “toroidal-pore,” or “carpet” mechanisms, primarily dependent on their sizes (Brogden, 2005). If a peptide's length is enough to span the entire bilayer of bacterial membrane, it will form barrel-stave pores where the peptide monomers form tight cylindrical bundles (e.g., Pardaxin, a 33-mer AMP from the fish *Pardachirus marmoratus*; Ramamoorthy et al., 2010). However, the intermediate-chain peptides (e.g., Magainin of 23-mer and Caerin 1.1 of 25-mer, two frog skin-derived AMPs) usually form toroidal pores with curved peptide-lipid edges (Murzyn and Pasenkiewicz-Gierula, 2003; Fernandez et al., 2009). For the peptides that are too short to span the entire bilayer (e.g., Aurein of 13-mer and Citropin 1.1 of 16-mer, two frog skin-derived AMPs), they often accumulate on the membrane surface and remain interfacially in contact with the lipid headgroups via the carpet model (Boland and Separovic, 2006; Fernandez et al., 2012). In the carpet model, higher peptide concentrations are usually required in the formation of lipid-peptide complexes to elicit membrane damage, providing an explanation for their relatively higher lethal doses when assayed in an *in vitro* condition. However, in the *M. eupeus* venom they may launch a joint action to commonly target bacterial membrane via the different mechanisms dependent on their sizes, namely the carpet model for Meucin-13, Meucin-18, MeuFSPL-1, and MeuFSPL-2 and the toroidal model for Meucin-22. A similar strategy might also be adopted by frogs because in their skins there are a large number of helical AMPs with different sizes to fight against skin infection (Vanhoye et al., 2003).

### Roles of svcAMPs in predation and defense

As generalist predators in their habitat, scorpions prey upon various insects, spiders, annelids, centipedes, mollusks, and other small animals. However, they are also victims from some vertebrate predators, such as mice, lizards and snakes. As a result, they evolved venoms for prey acquisition and defense against predators borne by neurotoxins affecting ion channels.

svcAMPs may also be participators of these two processes provided that tissue-specific AMPs often carry functions associated with tissues they exist. For example, Hepcidins, human liver-expressed AMPs, can function as iron-regulatory hormones targeting membrane-associated ferroportin (Michels et al., 2015); β-defensin 15 and Bin1, two epididymis-specific AMPs, are required for initiation of sperm maturation, motility and male fertility (Zhou et al., 2004; Zhao et al., 2011). Similarly, svAMPs, as venom-specific antibacterial agents, also evolved several venom-related functions: (1) Insect toxicity for predation; (2) Hemolysis, and (3) Pain induction for defense. The observation that mice injected with marmelittin showed similar symptoms of redness and pain to mice stinged by scorpion indicates that these hemolytic AMPs are the effector molecules of venom-induced pain and have ability to repel predators. In fact, this defensive strategy is also employed by other small venomous arthropods when they face physically larger vertebrate predators (Kuhn-Nentwig et al., 2011). Because these svcAMPs are capable of destroying blood cells and causing general tissue damage, they represent a new class of hemotoxins involved in defense of scorpions through evoking pain.

Although they exist in low abundance that appears to excludes their defensive and predatory functions alone, these svcAMPs could act as a synergist of neurotoxins. Recently, it was found that some spider toxins were able to elicit immediate and robust nocifensive responses (acute pain) through activating Na_v_1.1 channels (Osteen et al., 2016). Considering that Na^+^ channel toxins from the *M. eupeus* venom (Zhu et al., 2012b) and the spider toxins both bind to identical channel receptor site (called site 3) and evoke similar pharmacological effect - prolonging channel inactivation, it is reasonable to infer that this class of scorpion neurotoxins also serve pain inducer to commonly exert their effect with the svcAMPs in repelling predators as defensive substances. Since these hemotoxins and the neurotoxins both are lethal on insects, they may also commonly exert anti-insect roles in prey acquisition.

### Prospect of svAMPs as anti-infective drugs

With the alarming rise in antibiotic resistance and the sporadic emergence of new pathogens, there is an urgent need for new antimicrobial agents. For example, polyvinylpyrrolidone (PVP)-capped silver nanoparticles and methanolic extracts of medicinal plants are being explored as new generation of antibiotics (Tiwari et al., 2016, 2017). Because of broad-spectrum antimicrobial activity against a diversity of pathogens even cancer cells, together with their interaction with multiple microbial targets reducing the risk of resistance, AMPs are also considered as a promising alternative of antibiotics for novel drugs (da Cunha et al., 2016). Several svcAMPs have been tested for their anti-infective capability in animal models (Zhao et al., 2009; Fan et al., 2011). Other authors also mentioned the therapeutic potential of svcAMPs based on their *in vitro* data (Cao et al., 2012; Arpornsuwan et al., 2014; Du et al., 2015). They assumed that these peptides could be good candidates for designing peptide antibiotics. While such possibility cannot be excluded, two intrinsic features seem to limit their ability as drugs. Firstly, these svcAMPs are highly toxic to mammalian blood cells due to their venom origin, as discussed previously. Although several strategies have been designed to improve the cell selectivity, these studies primarily focused on structural modifications of svcAMPs through point mutations or deletions (Rodríguez et al., 2014; Wu et al., 2014; de la Salud Bea et al., 2015). These changes often led to peptides with enhanced antimicrobial activity but accompanying increased hemolysis (de la Salud Bea et al., 2015). In addition, several modified peptides showed enhanced antimicrobial activity and reduced hemolysis, but their stability was not evaluated (Rodríguez et al., 2014). Secondly, most svcAMPs show serum unstability when isolated from the venom, which is presumably due to the loss of protection from protease inhibitors in the venom. In our study, only MeuTxKβ-1 showed some potential as a drug lead in view of its low hemolysis, acceptable serum stability, and selective toxicity on several antibiotic resistant *Staphylococci* clinical isolates at low micromolar concentrations. In spite of these advanges, its toxicity on mammalian K^+^ channels needs to be further investigated.

## Conclusion

**Figure 10:**
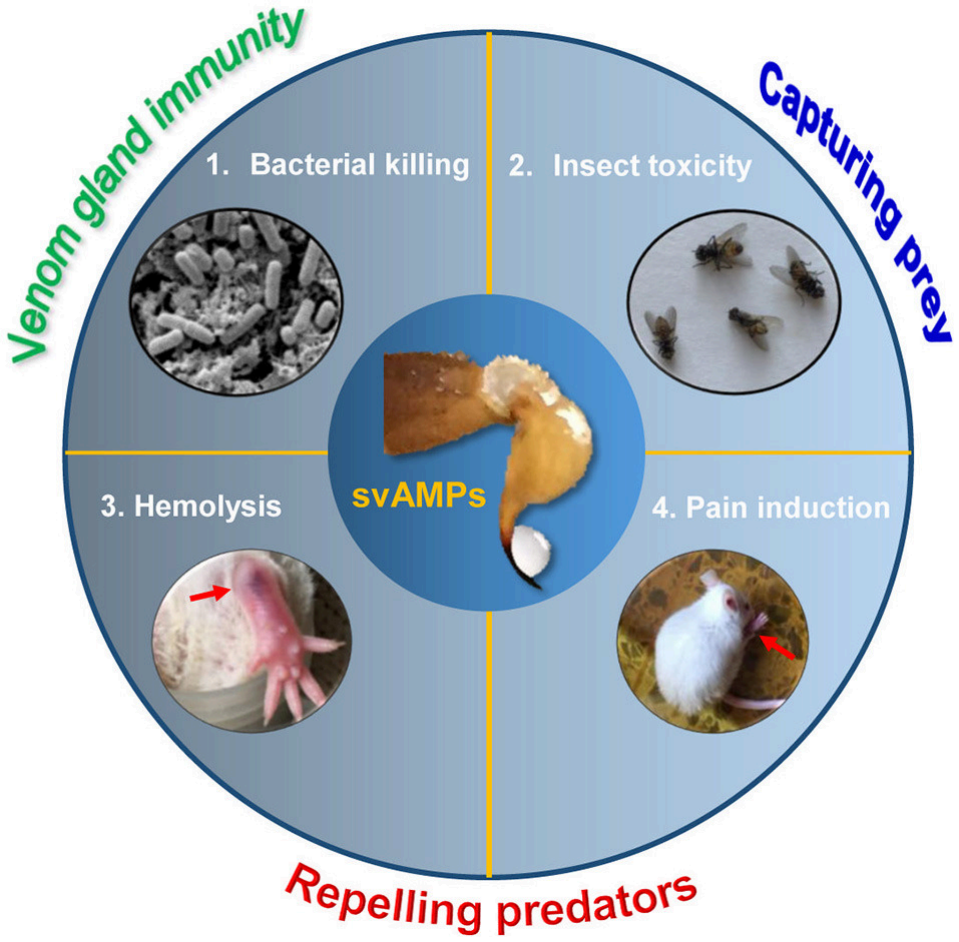
The biological functions of svAMPs. These include bacterial killing, insect toxicity, hemolysis and pain induction, which are involved in predation and defense of scorpions against both predators and microbes. The crude venom was extracted from the *M. eupeus* telson by electrostimulation.

This report reveals a broader variety of biological activities of svAMPs than first envisaged (Figure 10). Given their origin in the venom, the multifunctionality observed here represents an intrinsic features of these peptides, likely evolved by natural selection from microbes, prey and predators of scorpions. Our studies suggest that animal venoms are a new evolutionary model to study how multifunctions are evolved in an ancient, functionally limited antimicrobial scaffold. This will definitely be useful in guiding engineering modification of svAMPs to improve their target selectivity. The discovery of the widespread involvement of multiple venom components in scorpion predation and defense will stimulate more studies on their synergistic mechanisms against microbes and competitors. This will lead to a better understanding of the biological functions of animal venoms.

## Data archiving

Nucleotide sequences obtained in this study have been deposited in the GenBank database (http://www.ncbi.nlm.nih.gov/) and their accession numbers are provided in Table S2.

## Author contributions

SZ conceived and designed this study. BG and SZ performed the experiments. They both jointly wrote this paper and agreed to submit it to Frontiers in Microbiology.

### Conflict of interest statement

The authors declare that the research was conducted in the absence of any commercial or financial relationships that could be construed as a potential conflict of interest.

## Abbreviations

3D: three-dimensional
AMP: antimicrobial peptide
BK: big potassium channel (also called large conductance calcium-activated potassium channel)
CD: circular dichroism
CFU: colony-forming unit
CSαβ: cysteine-stabilized α-helix and β-sheet
FSPL: frog skin peptide-like
INDEL: insertion/deletion
LD_50_: half maximal lethal dose
MALDI-TOF MS: matrix-assisted laser desorption/ionization time of flight mass spectrometry
MRSA: methicillin-resistant *Staphylococcus aureus*
MW: molecular weight
PC: proprotein convertase
PI: propidium iodide
PRSA: penicillin-resistant *Staphylococcus aureus*
RP-HPLC: reversed-phase high-performance liquid chromatography
RT: reverse transcriptase
SD: standard deviation
SEM: scanning electron microscopy
SNP: single nucleotide polymorphism
svAMP: scorpion venom antimicrobial peptide
TFA: trifluoroacetic acid
TFE: trifluoroethanol.
