# Supplementary material for: Mesobuthus Venom-Derived Antimicrobial Peptides Possess Intrinsic Multifunctionality and Differential Potential as Drugs
Source: Front Microbiol. 2018 Feb 27;9:320. doi: 10.3389/fmicb.2018.00320 (PMC5863496; doi:10.3389/fmicb.2018.00320)
Supplement: Supplementary file 1 [file Table1.DOCX]

**Table S1. Primers used in this study**

**Name Sequence (5’→3’)**

FSPL-F CCAGAATATTCGAAACTCGGC

FSPL-R GAGAGACTAACGACTGTAGTA

MeVAMP-9-F  TCGAAGAAAATTCTATTCCAGT

MeuFSPL-F AGTTTCCGAAATCCTCCAGT

**Note: K, G or T; R, A or G; Y, T or C.**
