# Supplementary material for: Mesobuthus Venom-Derived Antimicrobial Peptides Possess Intrinsic Multifunctionality and Differential Potential as Drugs
Source: Front Microbiol. 2018 Feb 27;9:320. doi: 10.3389/fmicb.2018.00320 (PMC5863496; doi:10.3389/fmicb.2018.00320)
Supplement: Supplementary file 2 [file Table2.DOCX]

**Table S2.** Genes described in this study

Gene name Species Accession No. Clone Cloning method [primer name]

*Marmelittin* *M. martensii*  AF159979 cDNA cDNA library

*BmKb1* (cNDA) [CAA] [BJ] *M. martensii*  KJ425581 cDNA PCR [FSPL-F/3AP]

*BmKb1* (gDNA) [CAA] [BJ] (Clone7) *M. martensii*  DQ988345 gDNA PCR [FSPL-F/FSPL-R]

*BmKb1* (gDNA) [CAA] [BJ] (Clone16) *M. martensii*  DQ988346 gDNA PCR [FSPL-F/FSPL-R]

*BmKb1* (gDNA) [CAA] [BJ] (Clone17) *M. martensii*  DQ988347 gDNA PCR [FSPL-F/FSPL-R]

*BmKb1* (gDNA) [CAA] [BJ] (Clone19) *M. martensii*  [DQ988348](http://www.ncbi.nlm.nih.gov/nucleotide/119370330?report=genbank&log$=nuclalign&blast_rank=2&RID=FCCGMK8B014) gDNA PCR [FSPL-F/FSPL-R]

*Marcin-22* *M. martensii* ADT89763 cDNA PCR [MeVAMP-9-F/3AP]

*Marcin-22-1* *M. martensii* ADT89762 cDNA PCR [MeVAMP-9-F/3AP]

*Marcin-22-2* *M. martensii*  ADT89764 cDNA PCR [MeVAMP-9-F/3AP]

*MeuFSPL-1* *M. eupeus*  DQ988344 gDNA PCR [MeuFSPL-F/FSPL-R]

*MeuFSPL-2*  *M. eupeus* EF445102 cDNA cDNA library

*Meucin-22-1*  *M. eupeus* ADT89761 cDNA PCR [MeVAMP-9-F/3AP]

Note: ”BJ”, Beijing.
