# Supplementary material for: Mesobuthus Venom-Derived Antimicrobial Peptides Possess Intrinsic Multifunctionality and Differential Potential as Drugs
Source: Front Microbiol. 2018 Feb 27;9:320. doi: 10.3389/fmicb.2018.00320 (PMC5863496; doi:10.3389/fmicb.2018.00320)
Supplement: Supplementary file 3 [file Table3.DOCX]

**Table S3.** Sources of the bacteria used in this study

**Strains Sources**

*Gram-positive bacteria*

*Bacillus cereus* CGMCC 1.1846 Center for Microbial Resources, Institute of Microbiology, Beijing, China

*Bacillus megaterium* CGMCC 1.0459 Center for Microbial Resources, Institute of Microbiology, Beijing, China

*Bacillus subtilis* CGMCC 1.2428 Center for Microbial Resources, Institute of Microbiology, Beijing, China

*Micrococcus luteus* CGMCC 1.0290 Center for Microbial Resources, Institute of Microbiology, Beijing, China

*Staphylococcus aureus* CGMCC1.89 MSSA Center for Microbial Resources, Institute of Microbiology, Beijing, China

*S. epidermidis* PSSE P1111 302^nd^ Military Hospital, Beijing, China

*Staphylococcus* MRCNS P1369 302^nd^ Military Hospital, Beijing, China

*S. aureus* MRSA P1374 302^nd^ Military Hospital, Beijing, China

*S. aureus* PRSA P1383 302^nd^ Military Hospital, Beijing, China

*S. aureus* MRSA P1386 302^nd^ Military Hospital, Beijing, China

*S. epidermidis* PRSE P1389 302^nd^ Military Hospital, Beijing, China

*Staphylococcus aureus*  Gifted by Dr. Jing Qi (Shandong Academy of Agricultural Sciences, Jinan, China)

(Strain J685,J698,J700 J706,J708,J710)

*Streptococcus sanguinis* ATCC 1.2497 Center for Microbial Resources, Institute of Microbiology, Beijing, China

*Streptococcus salivarius* ATCC 1.2498 Center for Microbial Resources, Institute of Microbiology, Beijing, China

*Streptococcus mutans* ATCC 1.2499 Center for Microbial Resources, Institute of Microbiology, Beijing, China

*Staphylococcus warneri* ATCC 1.2824 Center for Microbial Resources, Institute of Microbiology, Beijing, China

*Streptomyces griseus* NBRC 13350 Center for Microbial Resources, Institute of Microbiology, Beijing, China

*Streptomyces scabiei* CGMCC 4.1765 Center for Microbial Resources, Institute of Microbiology, Beijing, China

*Gram-negative bacteria*

*Alcaligenes faecalis* CGMCC 1.1837 Center for Microbial Resources, Institute of Microbiology, Beijing, China

*Escherichia coli* ATCC 25922 Center for Microbial Resources, Institute of Microbiology, Beijing, China

*Escherichia coli*  Preserved by our own lab

(Strain DH5α, JM109, Top10)

*Escherichia coli*  Gifted by Dr. Jing Qi (Shandong Academy of Agricultural Sciences, Jinan, China)

(Strain Am. J16c, Am. J23a, CIP. J14b, D. G2b, D. J45b)

*Pseudomonas aeruginosa*  Gifted by Dr. Luyan Ma (Institute of Microbiology, Beijing, China)

(Strain O1, 14, FRD1)

*Pseudomonas aeruginosa* 374 Gifted by Prof. Yang Wang (China Agricultural University, Beijing, China)

*Pseudomonas aeruginosa*  Gifted by Dr. Jing Qi (Shandong Academy of Agricultural Sciences, Jinan, China)

(DH, 11092304, 11082616, QT1, 11092618, 11082603)

*Pseudomonas solanacearum* Gifted by Prof. Fengming Song (Zhejiang University, Hangzhou, China)

*Salmonella enterica* ATCC 14028 Center for Microbial Resources, Institute of Microbiology, Beijing, China

*Serratia marcescens* ATCC 14041 Center for Microbial Resources, Institute of Microbiology, Beijing, China

*Stenotrophomonas maltophilia*  Center for Microbial Resources, Institute of Microbiology, Beijing, China

(strain CGMCC 1.1788)

*Filamentous fungi*

*Aspergillus nidulans* A28 Prof. Shaojie Li, Institute of Microbiology, Beijing, China

*Aspergillus nidulans* Rcho15 Prof. Jae-Hyuk Yu, University of Wisconsin, Madison, WI 53706, USA

*Geotrichum candidum* CCTCC AY 93038 China Center for Type Culture Collection, Wuhan University, Wuhan, China

*Yeasts*

*Canidia albicans* Prof. Fengyan Bai and Prof. Guanghua Huang, Institute of Microbiology, Beijing, China

(JX1195, JX1009, JX1016, JX1017, 4247, 4257, 4259, 4277, SZ56, SZ72)

*Pichia pastoris* X33 Prof. Wenjun Liu, Institute of Microbiology, Beijing, China

*Saccharomyces cerevisiae* CCTCC AY 92003 Center for Microbial Resources, Institute of Microbiology, Beijing, China
