# Supplementary material for: Mesobuthus Venom-Derived Antimicrobial Peptides Possess Intrinsic Multifunctionality and Differential Potential as Drugs
Source: Front Microbiol. 2018 Feb 27;9:320. doi: 10.3389/fmicb.2018.00320 (PMC5863496; doi:10.3389/fmicb.2018.00320)
Supplement: Supplementary file 4 [file Image1.PDF]

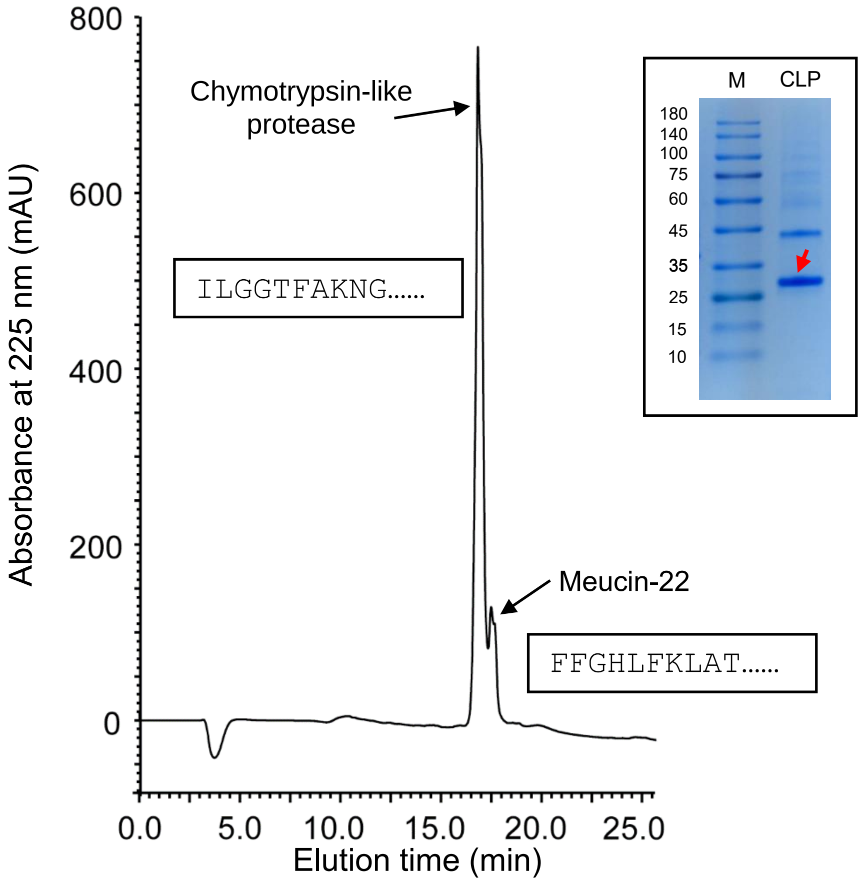

Fig. S1. **Repurification of Meucin-22 by RP-HPLC.** The Agilent Zorbax 300SB-C18 (4.6x150 mm, 5 μm) was equilibrated with 0.05% TFA in water (v/v), and peptide components were eluted from the column with a gradient from 0 to 20% acetonitrile within 3 min, 20%-80% acetonitrile within 20 min, 80%-100% acetonitrile within 0.1 min, with a flow rate of 0.6 ml/min. Meucin-22 was well seperated from two co-eluted proteins that had molecular masses of 28,069.18 Da and 28,215.46 Da, respectively, as detremined by the Agilent 6530 Accurate-Mass Q-TOF LC/MS system (USA) (data not shown), consistent with the result of SDS-PAGE (indicated by a red arrow. MW ≈ 28 kD, see the inset). Using Edman degradation, we showed that these two proteins had an identical N-terminal amino acid sequence (boxed) to two chymotrypsin-like proteases (CLPs) identified by cDNA cloning (ABR21066.1 and ABR21040.1). However, molecular weights (27027 Da and 26986 Da) of these two enzyme-like proteins calculated from their sequences are lower than the purified proteins, suggesting that these proteases might have been modified after post-translation.
