# Supplementary material for: Mesobuthus Venom-Derived Antimicrobial Peptides Possess Intrinsic Multifunctionality and Differential Potential as Drugs
Source: Front Microbiol. 2018 Feb 27;9:320. doi: 10.3389/fmicb.2018.00320 (PMC5863496; doi:10.3389/fmicb.2018.00320)
Supplement: Supplementary file 5 [file Image2.PDF]

Meucin-13	IFGA**I**AGLLKN**I**F[a]
Meucin-18	FFGH**L**FKLATK**I**IPSLFQ
MeuFSPL-1	FLFSL**I**PSAISG**L**ISAFK[a]
MeuFSPL-2	FLFSL**I**PSAISG**L**INAFK[a]
Meucin-22	FFGH**L**FKLATK**I**IPSLFQRKKE
Marcin-22	FFGH**L**FKLATK**I**IPSFFRRKNQ
Marmelittin	FLFSL**I**PSAISG**L**ISAFKGRRKRDLN[a]

Fig. S2. **Linear α-helical peptides used in this study.** The leucine/isoleucine residues presumably forming a short leucine zipper-like motif are boxed and marked in *bold*.
