# Supplementary material for: Mesobuthus Venom-Derived Antimicrobial Peptides Possess Intrinsic Multifunctionality and Differential Potential as Drugs
Source: Front Microbiol. 2018 Feb 27;9:320. doi: 10.3389/fmicb.2018.00320 (PMC5863496; doi:10.3389/fmicb.2018.00320)
Supplement: Supplementary file 6 [file Image3.PDF]

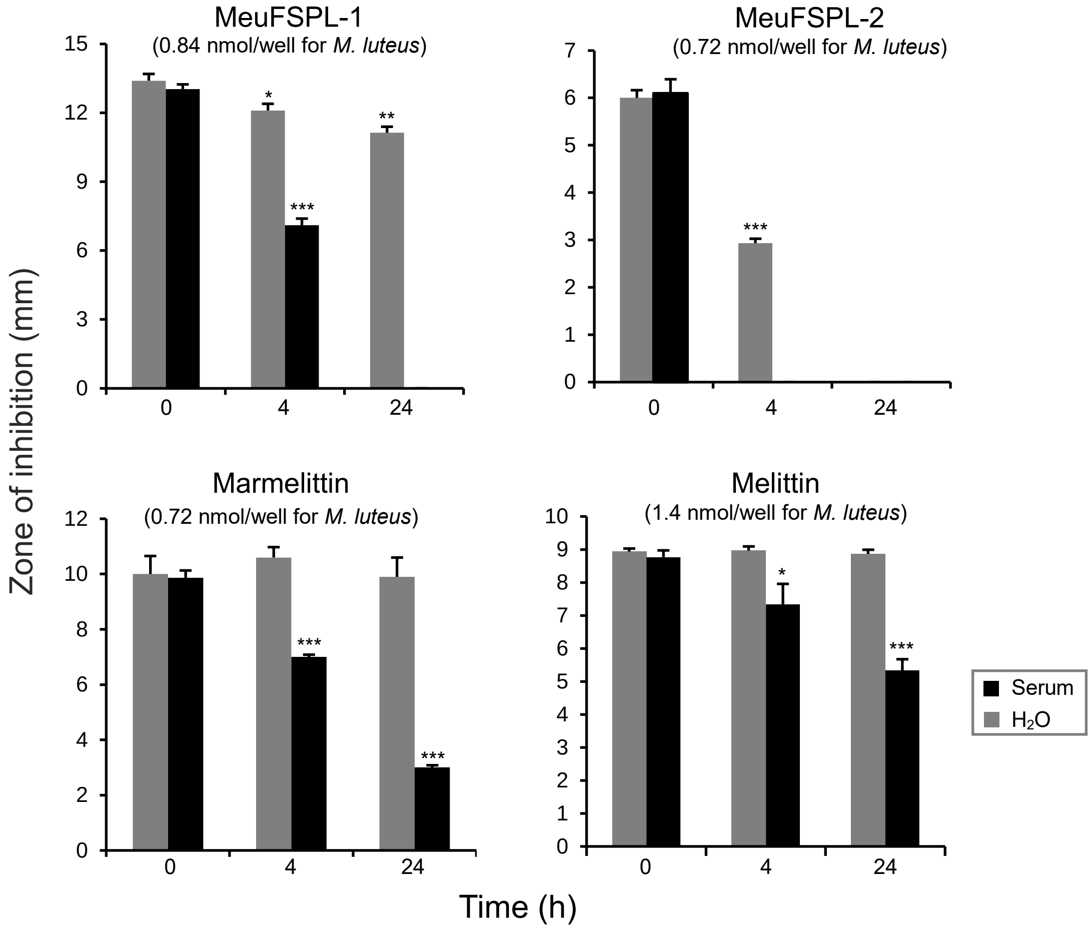

Fig. S3. **The stability of svcAMPs.** Peptides were incubated in H_2O or mouse serum for the indicated times and then added to *Micrococcus luteus* plates. Peptide doses used were 0.72-1.44 nmol each well dependent on their potency. Diameters of inhibition zones were recorded after incubation at 37ºC overnight. All experiments were performed in triplicate, and data are presented as mean ± standard deviation. **P < 0.01; ***P < 0.001 (compared with the control without incubation in H_2O or the serum).
